# A Computational Challenge of Guanine Quadruplex Involvement in Anticancer Antibiotics

**DOI:** 10.3390/ijms27083504

**Published:** 2026-04-14

**Authors:** Snezhana M. Bakalova, Nikoleta Kircheva, Silvia Angelova, Jose Kaneti

**Affiliations:** 1 Institute of Organic Chemistry with Centre of Phytochemistry, Bulgarian Academy of Sciences, 1113 Sofia, Bulgaria; snezhana.bakalova@orgchm.bas.bg; 2 Institute of Optical Materials and Technologies “Acad J. Malinowski”, Bulgarian Academy of Sciences, 1113 Sofia, Bulgaria; nkircheva@iomt.bas.bg (N.K.); sea@iomt.bas.bg (S.A.); 3 University Centre on Tautomeric Research and Education in Science and Technology (ERA Chair UCTREST), University of Plovdiv, 4000 Plovdiv, Bulgaria

**Keywords:** antibiotics, guanine quadruplexes G4, stacking, ONIOM computing

## Abstract

Small bioactive molecules show significant propensity to form noncovalent addition complexes with guanine quadruplexes, G4. The stabilization energies of these complexes have been computed precisely at the sufficiently high 6-31G** basis set level of density functional quantum chemical theory, DFT. A decisive factor in present model computations is the adopted size of G4 models, whether these consist simply of stacked quanine quartets, or also involve (deoxy)ribose-phosphate fragments of proper nucleic acids. The challenge is in the preservation of physico-chemical accuracy of DFT computations with increasing sizes of models, involving upwards of 120 atoms for the simplest two-layer G4, plus at least 60 pentose-phosphate linker atoms per each pair of guanine quartets. Bioactive ligand sizes add to the requirements for further rigorous analyses of the roles of G4 complexes in biological processes, which thus remain necessarily open-ended.

## 1. Introduction

**Figure 1:**
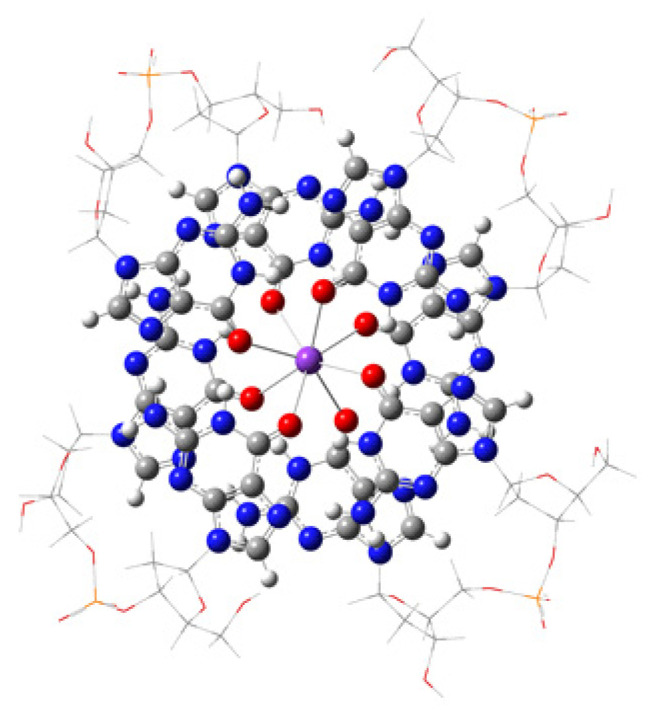
The model quadruplex, with the approximate space-centered anticube around the purple K^+^, with the 8 red oxygen atoms of packed guanines. Nitrogen atoms are blue. Grey balls are carbons, and small white balls are hydrogens. 2-Deoxyribose phosphate linkers are shown as wireframes for the ONIOM calculations. Atoms at wireframe angles are colored correspondingly. Phosphorus is orange. Visualization: Gauss View [17].

By the time of their discovery, G-quadruplexes (G4s), secondary structures in DNA or RNA, had been considered “unusual” or “noncanonical”, but after several decades of intensive studies, they are now known to be integral constructs within the nucleic acids helices [1]. In recent years, some useful, although not exhaustive, reviews on this hot topic have been published [2,3,4,5,6]. Moreover, in the ONQUADRO database [7], experimentally determined G-tetrad, quadruplex, and G4 helical structures are being collected from nucleic acids deposited in the protein database (PDB) obtained by X-ray diffraction or NMR. The composition of G-quadruplexes is quite distinctive. Repetitively expressed guanines folded in G4 sequences self-assemble into stacked G-tetrads characterized by their square planar arrays tightly stabilized by guanine–guanine Hoogsteen hydrogen-bonding. The phosphodiester backbone spirals around the tetrad core, while a metal cation—most commonly potassium K^+^—occupies the apparent center of the apparent anticube of O_6_ atoms of the eight guanine bases of each pair of quartets [8], see Figure 1. Occasionally, several other metal ions such as sodium (Na^+^), rubidium (Rb^+^), cesium (Cs^+^), strontium (Sr^2+^), thallium (Tl^+^), calcium (Ca^2+^), lead (Pb^2+^), and barium (Ba^2+^) are found to be able to stabilize quadruplexes [9], with a number of studies estimating the general G-quadruplex stabilization trend: Sr^2+^ > K^+^ > Ca^2+^ > NH_4_^+^, Na^+^, Rb^+^ > Mg^2+^ > Li^+^ ≥ Cs^+^ [10]. We have also studied, by computational methods, the stabilization of our model two-layered quadruplex by K^+^, Na^+^, and Sr^2+^ [11]. On the other hand, X-ray crystallographic analyses, along with several multinuclear NMR studies, have been able to directly locate only Na^+^, K^+^, Rb^+^, and Ca^2+^ ions, naturally occurring in or between the tetrads [12]. The metal cation plays indeed a crucial role in the stabilization of the G-quadruplex architecture, as demonstrated by the charge transferred from the potassium cation to the coordinating eight O_6_-atoms internal of the tetrads. A Hirshfeld analysis [13] conducted at the wB97XD/6-31+G(d,p) level, see Figure S1, provides solid proof for the ion–dipole interaction, where the estimated charge of the monocationic K^+^ is found to be 0.89 e^−^ in the ‘pure’ metal-GG complex. Note that this outcome, along with potassium’s extremely high cellular concentration and suitable ionic radius, contributes to a great extent in specifically choosing K^+^ among all other metal cations present in the cellular milieu for stabilizing the ion channel in the quadruplex structure. The combined use of several spectroscopic and biophysical techniques, such as UV, fluorescence [14], and circular dichroism spectroscopy [15], optimally together with X-ray crystallographic [16] and/or NMR analyses, validates the existence of a stable quadruplex.

G-quadruplexes’ more frequent presence in cancer tissues, as compared to normal ones [8,16], along with their over-expression in cancer-promoting genes [18,19,20], associates them with processes and control mechanisms that play a key role in the biology and growth of malignant formations. Hence, it is beneficial to consider G4s in their role as targets of immense potential in anticancer therapy. For example, the G-Quadruplex Ligands Database (http://www.g4ldb.com/) [21] collects and deposits small molecules targeting G4 structures. These ligands are distinguished by having a planar aromatic fragment for π-π stacking with G-tetrads, bulky groups sterically preventing intercalation with double-stranded DNA, and a positive charge or lone electron pair substituents for binding to grooves or loops [22].

**Figure 2:**
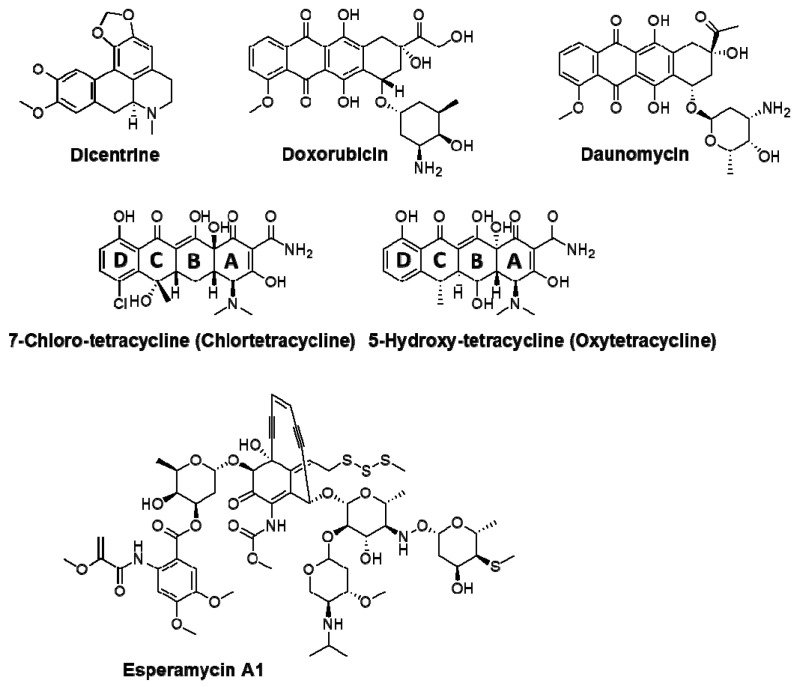
Standard stereochemical drawings of antibiotic structures considered in this study. Also see Figure S2.

An intriguing class of anticancer drugs holding great potential comprises the antineoplastic or antitumor antibiotics [23,24], with the anthracycline antibiotics, e.g., doxorubicin, among the most effective agents. In the current study, the possible complexation of some interesting anticancer antibiotics to a G-tetrad model is to be assessed. Their chemical structures are drawn in Figure 2. We have decided to include representatives of different classes, namely an aporphinic alkaloid (dicentrine), two of the most potent aminoglycoside antibiotics (doxorubicin and daunomycin, also known as daunorubicin), two tetracyclin antibiotics (7-chloro-tetracyclin and 5-hydroxy- tetracyclin), and esperamycin A1, a chromoprotein enediyne antitumor antibiotic of bacterial origin, and one of the two most potent antitumor agents known. The main purpose is to draw a more clear picture of the G4 quadruplex–ligand interactions based on the different structures of antibiotics. Therefore, the most crucial part of the model computations focuses on the energy surface encompassing the quadruplex and the antibiotic, which has been calculated at the highest theoretical level practically available to us (See Computational details). For this particular reason, in the case of esperamycin, only the smallest representative of the group, A1, see Figure 2, has been included in the modeling process.

The present study comes as a continuation of our previous efforts to contribute to the understanding of G-quadruplexes’ intricate biology and their interaction with diverse ligands by applying the powerful tools of computational chemistry. Our first stumble with G4 tentatively giving explanations of heterocyclic ligand activity in suppressing cell proliferation came from a study of synthetic quinazoline derivatives [25]. Literature data apparently supported direct interaction of heterocyclic ligands and G4, which we modeled as a stacking interaction of guanine quadruplexes with the respective π-electronic heteroaromatic system [25]. Moreover, directed synthesis of a series of nitrogen heterocycles provided, somewhat unexpectedly, a linear quantitative structure–activity relationship, a QSAR-type relationship between their computed DFT stacking affinities to a “naked” G4 model, and the respective log(IC_50_) for a melanoma cancer cell line [26]. The simplest “naked” G4-model at this initial stage consisted of two stacked guanine quartets, at ca. 3.30 Å of each other, rotated 45° with respect to each other, and with an alkali cation, K^+^ or Na^+^, in the apparent center of symmetry between the layers. The computed distance between two guanine quartets is close to the step size, 3.40 Å, of the quadruple helix [27] of polyguanilic acid. The relative success of the mentioned initial models encourages further detailed studies of G4s themselves, as well as their interactions with potential ligands. A number of studies involving the pentose-phosphate linkers of guanine quartets [27], treated beyond the level of molecular mechanics, set the approximate borderline, around 400 atoms, between small and larger supramolecular systems [28]. To pursue possible complexes of antibiotics and G4, with sizes about and above the mentioned number of atoms, we will certainly need a robust computational approach. Our choice here will be based on the ONIOM concept of Morokuma and coworkers [29], with the decision to treat all fragments of the envisaged supramolecular complexes of G4 and antibiotics, the former primitive model [24], at a high quantum chemical DFT level. Pentose-phosphate linker fragments, encompassing the G4 “core”, will be considered at a quantum chemical level as well, utilizing the semiempirical PM7 approximation [29,30].

## 2. Results and Discussion

For better compatibility of results, energetic parameters of the studied interactions are collected in Table 1. Q2K is the model two-layered quadruplex; oni_Q2 is Q2K plus the 2-deoxy-ribose stem fragment, used as the low-level layer in ONIOM, see Figure 2. From ONIOM runs, only the high-level ωB97XD/6-31G** energies are given.

### 2.1. Dicentrine–G4 Interaction

**Figure 3:**
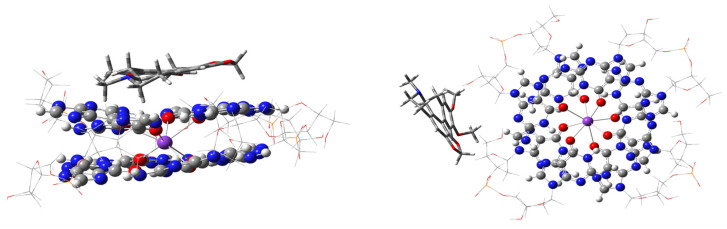
Optimized geometries of ONIOM stacking and “external groove”, or out-of-G4-plane, modes of interaction of dicentrine with a two-layered G4. The high wB97XD/6-31G** level of calculation, used for G4 and the ligand, is visualized as balls and sticks for G4, while the ligand is shown as sticks only. The low-level (PM7) fragments are shown as wireframe around guanine quartets, GaussView [17]. Atom color codes are C—gray; N—blue; O—red; K—purple, P—orange. Ribose-phosphate atoms are at the corners of the wireframe.

An initial example of computed interaction modes of a natural alkaloid, dicentrine, with the IUPAC name (7aS)-10,11-dimethoxy-7-methyl-6,7,7a,8-tetrahydro-2H,5H-benzo[g][1,3] benzodioxolo[6,5,4-de]quinoline [31], is a good illustration of the undertaken study. Figure 3 shows our two main modes of ligand–G4 interaction, the stacking mode with a guanine quartet on the left, and an “external” mode, which may be related to ligand attachment to a loop or groove of the G4 quadruple helix [31,32]. We are aware of the possible existence of a much larger set of minima on the potential energy surface of ligand–G4 interactions and may only relate these to each other in some order of preference. Experimental evidence has been interpreted as an indication of preferential binding of dicentrine to grooves of telomeric quadruplexes [32], while MD simulations show a preference for a stacking arrangement to a guanine quartet [32].

With the first example of G4–dicentrine interaction, we find a clear preference for the stacking ligand arrangement, where the electron correlation interaction of ligand–G4 π-systems brings stabilization of the stacked complex. The groove complex model, to the contrary, looks even destabilized with respect to isolated components by several kcal/mol. This result is totally out of line with reported experimental data so far [30], claiming that dicentrine does indeed stabilize the G-quadruplex structure in telomeres of tumor cells, thus promoting their apoptosis. Moreover, the complexation energies presented in Table 1 bring further theoretical evidence, as the values stand firmly on negative ground, being −41.1/−41.3 kcal/mol, depending on the applied methodology. Note that the Q2K@Dicentrine complex corresponds to the ‘stacked’ geometry. Therefore, our results support the claim expressed in Ref. [31] that the alkaloid might exert a potential anticancer effect and hence be considered a potential compound for targeting G-quadruplex structures localized at the telomeres. The results reported in Table S1 for the complexation energies in two solvents—n-octanol and water—do not differ substantially from those in the gas phase and stay close, amounting to −36.7/−35.3 kcal/mol.

### 2.2. Anthraquinone Antibiotics–G4 Interaction

**Figure 4:**
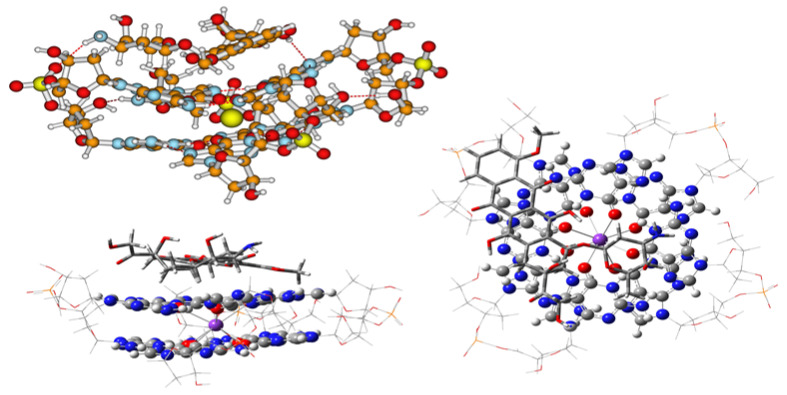
Stacking of doxorubicin to a G4 in the deoxy-ribose-phosphate envelope; top left: full ωB97XD in vacuo; other images are ONIOM (ωB97XD:PM7) in water, CPCM. The most prominent golden atom on the top left is K^+^ {MOLDEN [37]}, Carbon atom are light brown, phosphorus and K are in different shades of golden. Visible are the hydrogen bonds (dotted red lines, top left) with red phosphate O and blue guanine N.

Complexes of anthraquinone antibiotics with G4 have apparently been the first G4 adducts for which X-ray structural data became available [33,34,35,36]. The observed actual complexes have been of the stacking type with one or two ligands arranged parallel to the G-quaruplex. Structures of these complexes may have been the very source of the notion that the preferential types of ligands to stack to G-quadruplexes will most likely be large aromatic heterocyclic systems [16]. For this reason, we shall discuss a larger group of antibiotics stacking to G4, possibly revealing their activity due to this very mechanism. Here, we turn our attention to a bunch of tetracyclic antibiotics [36], with the intent to identify whatever differences might exist in their interactions with G4, see Figure 4, and beyond.

**Figure 5:**
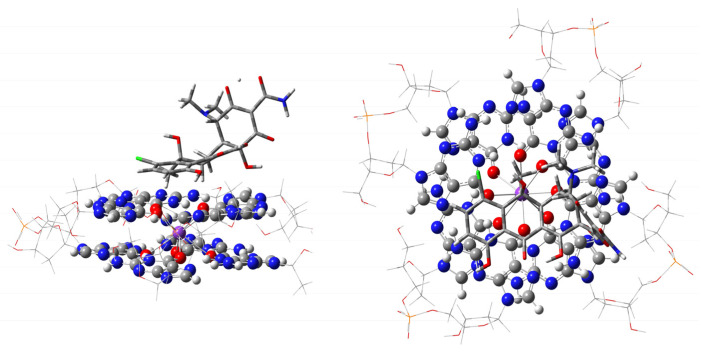
The stacking complex of 7-chloro-tetracycline with G4. Chlorine is green.

**Figure 6:**
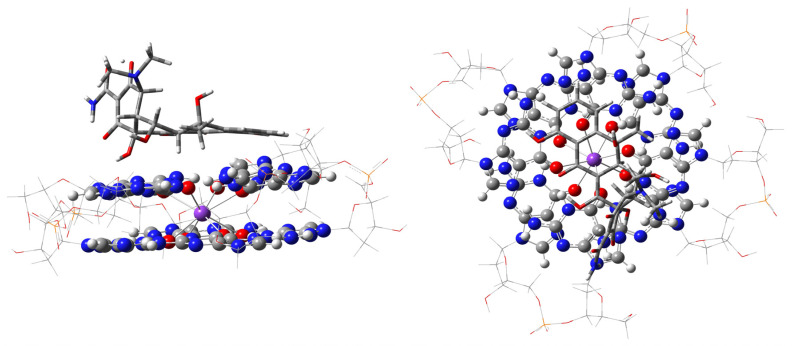
The stacking complex of 5-hydroxy-tetracycline and G4.

Several complexes of similar general view are expectedly produced from computations of tetracyclines [36] with the model G4. Complexes of the popular 7-Cl and 5-OH tetracyclines are shown in Figure 5 and Figure 6. It should be noted, preliminarily, that tetracyclines are predominantly ribosome-binding antibacterial agents, rather than direct participants in DNA replication processes.

**Figure 7:**
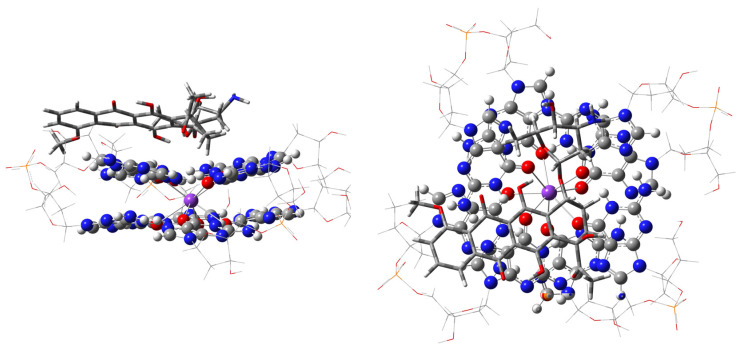
The complex of daunomycin with the model G4.

The difference in the complexation energies of the two tetracyclines in Table 1 may well be due to the difference in their arrangement. In the first case, the tetracycle remains largely planar and is located over just a pair of guanines. The 7-Cl substituent preserves the aromatic D-ring planar, while the 5-OH introduces a new chiral center in the B-ring, Figure 2, and the tetracycle with potentially drastically changed conformation has to move to the diameter of the top guanine quartet. For completeness, we also show the complex of a classical anthraquinone antibiotic, daunomycin, in Figure 7.

The calculations bring further theoretical evidence that the antibiotics under study can interact with the G4 quadruplexes by effectively stacking on their surface. The obtained complexation energies reported in Table 1 stand firmly in the negative region. Notably, the results for the more potent doxorubicin and daunomycin [33,34] are about 10 kcal/mol more in absolute value as compared with the tetracycline derivatives, which corresponds well to experimental data reported in the literature [35,36].

### 2.3. Esperamycin Antibiotics–G4 Interaction

**Figure 8:**
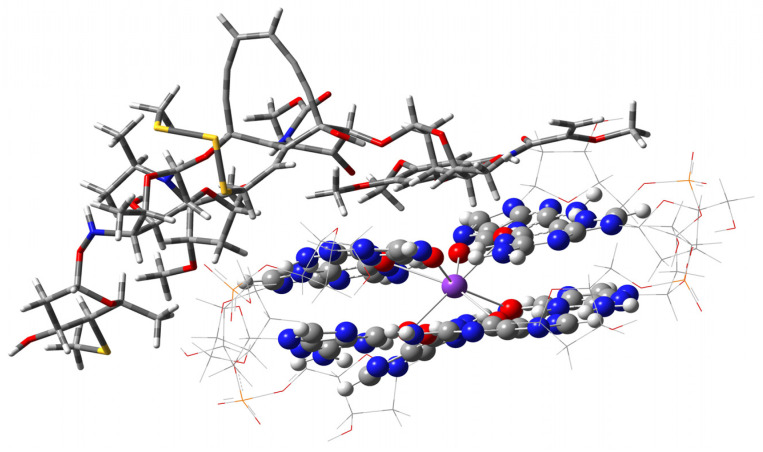
An unlikely “friendly” approach of esperamycin A1 to G4. The meaning is that all reactive fragments, enediyne ten-membered cycle on top, next to the allyl-trisulfide group in yellow, and NH-O, left, of the antibiotic are directed away from G4 in the preliminary manual approaching of the drug, as well as G4’s own glycosidic fragments, shown as the wireframe.

As demonstrated in the above Figures and Table 1, the absolute complexation energies of various ligands to G4 may vary anywhere up to ca. −59.0 kcal/mol for the most potent, doxorubicin, which is indicative of possible significant conformational changes in helical quadruplex structures and plausible antibiotic–quadruplex interaction [38]. These changes may well be indicative of very broad affinities of G4 to various agents, contacting their quadruplex helical fragments and their relatively low specificity with respect to diverse chemical agents. More examples in this direction may be given with antibiotics acting on their (deoxy-)ribose phosphate linkers, as shown with esperamycin antibiotics. This class of compounds is shown to directly bind to the minor groove of DNA due to its high hydrophilicity, and reacts further due to its ene-diyne fragment and allyl-trisulfide group [38,39], exactly by breaking NA ribose-phosphate strands following diradicaloid mechanisms. In Figure 8, we show an optimized “friendly” approach of esperamicin-A_1_ to our model G4, that is, with the quinonoid fragment stacked to the guanine quartet plane. The intended dual purpose is to point out once again the existence of multiple minima on the studied multidimensional interaction energy surfaces, as well as to underscore the arising problems of ligand (or drug, in this specific case) selectivity, related to computational searches of novel therapeutic agents, even with a preselected plausible mechanism of action.

The interaction energy of esperamycin A and G4 is rather meager and much lower in absolute value as compared to the other studied antibiotics, or even the alkaloid dicentrine, amounting to −23.2 kcal/mol. This probably means that the mode of interaction of esperamycin with DNA should not count on antibiotic stacking to G4, contrary to the case with doxycycline and even tetracyclines. A different preliminary selection of the starting point of optimization, with the reactive enediyne fragment approaching G4, crashes with uncontrollable mixing to glycosidic parts of the quadruplex, in line with the experimentally proven mechanism of esperamycin–DNA interaction [38,39]. The reason for this behavior could also be easily found in the obviously great internal freedom of esperamycin for conformational changes [40], which is a basic factor precluding the specific G4 stacking type of pharmacological activity. Nevertheless, as far as the structure of a drug involves a relatively big π-electron dominated fragment, we believe that the G4 stacking interaction might be of use in the understanding of its mechanism.

## 3. Materials and Methods

Our early model calculations of G4 use DFT with the 6-31G** split-valence double-zeta Gaussian basis set. Critical parts of these results are shown in Tables S1–S3. Our choice of functional capable of adequate description of hydrogen bonding as well as quartet stacking interactions has been the ωB97XD [41], which allows accurate description of long range interactions, including dispersion, expected in relatively large guanine quartet fragments, as well as of the stacking π-interactions of the latter. Software programs used in this study are Gaussian 16 [42] and ORCA, releases 5.0 and 6.0 [43,44]. Pure DFT runs used both programs, while ONIOM (ωB97XD: PM7) [30,45] are only run under Gaussian. The choice of computational level for the fragments in used ONIOM models is high (wB97XD/6-31G**) for G4 and ligands, while the low PM7 level is used for the anti-parallel deoxyribose-phosphate linkers [29,30]. Occasionally, badly converging supramolecular model geometries have been run preliminarily at the semiempirical PM7 quantum chemical level, using MOPAC [46]. Problems with slow convergence of ONIOM large scale runs occasionally forced us to loosen their final convergence criteria. Solvent effects on complex formation have been studied by single point calculations in two solvents, 1-octanol (ε = 10) and water (ε = 78) within the CPCM formalism [29,30]. The problem with the large sets of possible multidimensional energy surface minima has been overcome by preliminary adjusting (placing and rotating) the ligands to G4 manually, using GaussView [17]. In this sense, all described computational minima have been preselected, and none is the result of random search. The reason is, mathematically, that solutions of multidimensional systems of differential equations are critically dependent on their corresponding starting approximations.

## 4. Conclusions

The idea of aromatic molecules, hydrocarbons, or heterocycles readily participating in electron exchange correlation interactions is old enough. Our efforts to understand the activity of aromatic heterocyclic compounds led us to the involvement of G4 as a great natural example of a layered macrocyclic π-electron system. As such, G4s are capable of manifestation of character-defining roles in numerous biological processes, beginning with the many processes and functions of nucleic acids, and taking part in all NA-controlled cell functions. G4 interaction energies with ligand molecules are readily computable and, as such, quantitatively define the physical and chemical parameters of observable cell processes. For a number of aromatic and quasi-aromatic antibiotics, the involvement of G4 seems obvious, and examples of their activity may be amenable to QSAR computational searches. There are indeed important exceptions, where structural and/or reactivity characteristics of antibiotics preclude their interaction with G4. The latter examples possibly require detailed mechanistic studies of biological pathways, as well as computational analyses of involved biochemical processes, rates, and equilibria.

## Figures and Tables

**Table 1:** Gas-phase energies of the computed quadruplex Q2K, ligands, and Q2K–ligand complexes (Q2K@Ligand), in Hartree (1 Eh = 627.51 kcal/mol). Complexation energies ΔE (ΔE = E(Q2K@Ligand) − E(Q2K) − E(Ligand)) are given in kcal/mol. ONIOM-calculated structures are indicated by superscripts.

Species	E	ΔE
Q2K^ONIOM^	−4939.409965	-
Dicentrine	−1130.063064	-
Doxorubicin	−1927.981834	-
Daunomycin	−1852.788626	-
Chlortetracycline	−2023.265595	-
Oxytetracycline	−1563.69834	-
Esperamycin A1	−5761.679936	-
Q2K@Dicentrine^ONIOM^	−6069.537735	−41.1
Q2K@Doxorubicin^ONIOM^	−6867.484673	−59.0
Q2K@Daunomycin^ONIOM^	−6792.285586	−56.1
Q2K@Chlortetracycline^ONIOM^	−6962.747174	−45.5
Q2K@Oxytetracycline^ONIOM^	−6503.172111	−40.6
Q2K@Esperamycin A1^ONIOM^	−10701.12594	−23.2
Q2K	−4939.424722	-
Q2K@Dicentrine	−6069.552726	−41.3
Q2K@Chlortetracycline	−6962.762174	−45.7
Q2K@Oxytetracycline	−6503.187111	−40.8

## Data Availability

The original contributions presented in this study are included in the article/Supplementary Material. Further inquiries can be directed to the corresponding author.

## Supplementary Materials

The following supporting information can be downloaded at: https://www.mdpi.com/article/10.3390/ijms27083504/s1.
