# Supplementary material for: A Computational Challenge of Guanine Quadruplex Involvement in Anticancer Antibiotics
Source: Int J Mol Sci. 2026 Apr 14;27(8):3504. doi: 10.3390/ijms27083504 (PMC13115669; doi:10.3390/ijms27083504)
Supplement: Supplementary file 1 [file ijms-27-03504-s001.zip › ijms-4203954-supplementary.pdf]

Supporting Information

A Computational Challenge of Guanine Quadruplex Involvement in Anticancer Antibiotics

Snezhana M. Bakalova [1], Nikoleta Kircheva [2,3], Silvia Angelova [2,3] and Jose Kaneti [1*]

[1] Institute of Organic Chemistry with Centre of Phytochemistry, Bulgarian Academy of Sciences, 1113 Sofia, Bulgaria
[2] Institute of Optical Materials and Technologies "Acad J. Malinowski", Bulgarian Academy of Sciences, 1113 Sofia, Bulgaria
[3] University Centre on Tautomeric Research and Education in Science and Technology (ERA Chair UCTREST), University of Plovdiv, Plovdiv 4000, Bulgaria
* Correspondence: jose.kaneti@orgchm.bas.bg

C O N T E N T S

1. **Figure S1.** Reduced density gradient (RDG) analysis of dicentrine in complex with a two-layered G-quadruplex (G4) **P. 2**
2. **Figure S2,** Optimized geometries at the wB97XD/6-31G** level **P. 3**
3. **Table S1**. Energies of the computed quadruplex Q2K, ligands, etc... **P. 4**
4. **Table S2.** Optimized geometries of ligands and complexes **P. 5**
5. **Table S3.** The computational workflow **P. 25**

S5.1. ChemSketch **P. 25**
S5.2. MOPAC input **P. 31**
S5.3. MOPAC archive **P. 34**
S5.4. GAUSSIAN input **P. 38**
S5.5. GAUSSIAN output (optimized coordinates, and archive) **P. 41**

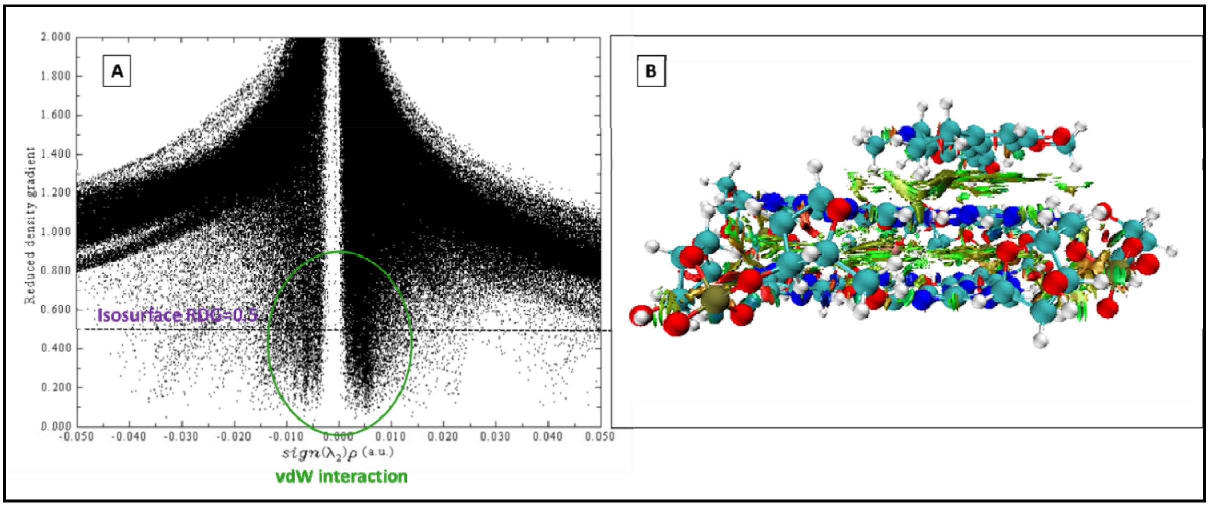

Figure S1. Reduced density gradient (RDG) analysis of dicentrine in complex with a two-layered G-quadruplex (G4). (A) RDG versus sign(λ_2)ρ plot highlighting noncovalent interactions, where negative values indicate attractive interactions (e.g., π–π stacking and hydrogen bonding), values near zero correspond to weak van der Waals interactions, and positive values represent steric repulsion. (B) Three-dimensional RDG isosurface at RDG = 0.5 mapped with a color scale (blue–green–red), where blue denotes strong attractive interactions, green indicates weak dispersive contacts, and red represents repulsive regions. The plots reveal that dicentrine binding to the G4 structure is predominantly stabilized by van der Waals interactions, while also involving attractive interactions such as π–π stacking and hydrogen bonding, along with regions of steric repulsion.

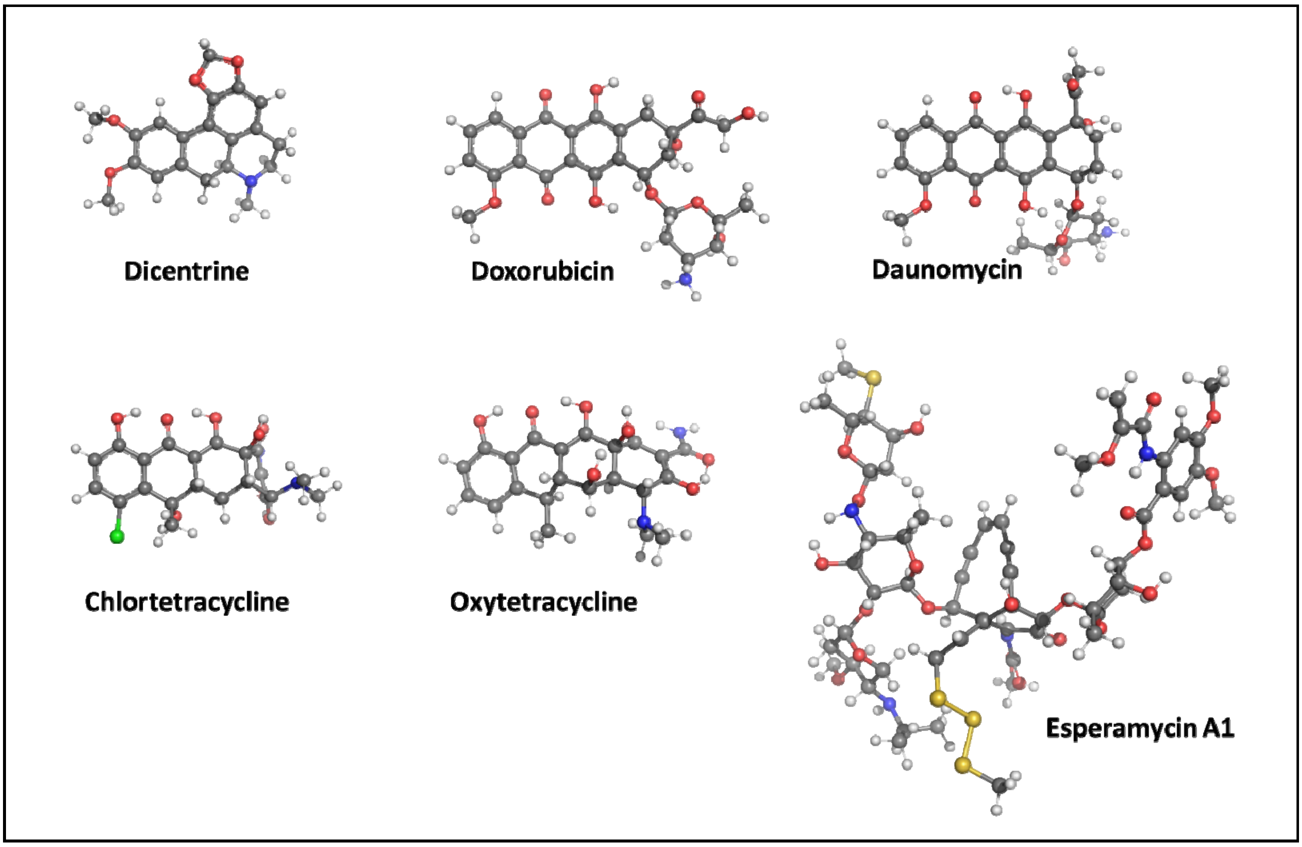

Figure S2. Optimized geometries at the wB97XD/6-31G** level of theory in the gas phase of antibiotics considered in this study. Their chemical structure is presented in Figure 1.

Table S1. Energies of the computed quadruplex Q2K, ligands, and Q2K–ligand complexes (Q2K@Ligand) in n-octanol and water environments, given in Hartree. Complexation energies ΔE (ΔE = E(Q2K@Ligand) − E(Q2K) − E(Ligand)) are reported in kcal/mol.

**Species**	**E**	**ΔE**
n-octanol		
Q2K	-4939.512555	-
Dicentrine	-1130.073547	-
Chlortetracycline	-2023.279974	-
Oxytetracycline	-1563.713437	-
Q2K@Dicentrine	-6069.642986	-36.3
Q2K@Chlortetracycline	-6962.857963	-41.7
Q2K@Oxytetracycline	-6503.281943	-35.7
water		
Q2K	-4939.522	-
Dicentrine	-4939.522299	-
Chlortetracycline	-1130.074735	-
Oxytetracycline	-1563.715077	-
Q2K@Dicentrine	-2023.281544	-35.7
Q2K@Chlortetracycline	-6962.868429	-41.1
Q2K@Oxytetracycline	-6503.292329	-35.1

Table S2. Optimized geometries of the ligands and selected Q2K–ligand complexes.

**Dicentrine**	6	2.983430000	0.597769000	0.414447000
	6	3.279213000	-0.726765000	0.042091000
	6	2.237564000	-1.573283000	-0.326260000
	6	0.916269000	-1.126119000	-0.327005000
	6	0.621154000	0.189299000	0.042732000
	6	1.672996000	1.038789000	0.411018000
	6	-0.211854000	-2.043991000	-0.720337000
	6	-1.429876000	-1.798917000	0.174317000
	6	-1.837397000	-0.336698000	0.042447000
	6	-0.789100000	0.624575000	0.014950000
	7	-2.526891000	-2.713079000	-0.137371000
	6	-3.740750000	-2.315698000	0.563505000
	6	-4.257224000	-1.003284000	0.002555000
	6	-3.174161000	0.045065000	-0.018633000
	6	-3.535564000	1.401213000	-0.115004000
	6	-2.520223000	2.321276000	-0.133830000
	6	-1.185012000	1.945920000	-0.069722000
	8	-2.612066000	3.680811000	-0.255997000
	6	-1.297438000	4.165746000	-0.030433000
	8	-0.401428000	3.070910000	-0.158108000
	1	-1.101724000	-1.955218000	1.226000000
	8	4.588496000	-1.081649000	0.085303000
	6	4.923284000	-2.406681000	-0.258676000
	8	3.974443000	1.445336000	0.830736000
	6	4.811841000	1.919237000	-0.211592000
	6	-2.193793000	-4.092425000	0.174825000
	1	2.438444000	-2.600925000	-0.608567000
	1	1.485572000	2.063688000	0.705268000
	1	-0.510771000	-1.871119000	-1.762464000
	1	0.127416000	-3.079460000	-0.644755000
	1	-4.493615000	-3.097956000	0.426535000
	1	-3.566359000	-2.219586000	1.653547000
	1	-4.622650000	-1.172390000	-1.017365000
	1	-5.108096000	-0.652784000	0.596858000
	1	-4.575027000	1.704446000	-0.172329000
	1	-1.053448000	4.922045000	-0.779402000
	1	-1.226045000	4.581348000	0.985352000
	1	4.435680000	-3.130551000	0.405844000
	1	4.655695000	-2.635142000	-1.298123000
	1	6.003918000	-2.486610000	-0.140709000
	1	5.348312000	1.097974000	-0.697678000
	1	4.228310000	2.468474000	-0.961528000
	1	5.530809000	2.596917000	0.251596000
	1	-1.907284000	-4.237469000	1.233781000
	1	-3.060775000	-4.725468000	-0.031951000
	1	-1.375839000	-4.452091000	-0.452159000
**Daunomycin**	6	2.335363000	2.355610000	1.924856000
	6	1.568102000	3.629876000	1.532349000
	6	0.847727000	3.551053000	0.166660000
	6	0.036896000	2.258201000	0.106725000
	6	0.643621000	1.093534000	0.574213000

	6	2.121285000	1.231238000	0.917509000
	6	-1.265594000	2.204584000	-0.416939000
	6	-1.974963000	0.991432000	-0.384303000
	6	-1.378379000	-0.176162000	0.139513000
	6	-0.082778000	-0.121054000	0.660572000
	6	-3.367537000	0.974792000	-0.836849000
	6	-4.190352000	-0.235352000	-0.577368000
	6	-3.596114000	-1.406174000	-0.089069000
	6	-2.105278000	-1.479882000	0.030787000
	6	-5.562659000	-0.151539000	-0.806986000
	6	-6.359513000	-1.247606000	-0.522219000
	6	-5.803206000	-2.414651000	-0.009943000
	6	-4.424596000	-2.511284000	0.208551000
	8	-3.838741000	-3.605550000	0.723991000
	6	-4.643060000	-4.710473000	1.073061000
	8	-1.514429000	-2.539154000	-0.027562000
	8	-3.889582000	1.965043000	-1.365973000
	8	0.415430000	-1.222984000	1.244252000
	8	-1.789229000	3.311299000	-0.950978000
	1	1.280772000	-1.023426000	1.636226000
	1	-2.704838000	3.082488000	-1.245398000
	6	-0.027545000	4.836725000	0.067046000
	8	-0.642236000	5.220890000	1.036106000
	6	0.033833000	5.620091000	-1.221238000
	8	1.776643000	3.489521000	-0.915030000
	1	2.442585000	4.170389000	-0.775128000
	8	2.724142000	0.055719000	1.440695000
	6	3.249057000	-0.807171000	0.453030000
	8	3.217136000	-2.068600000	1.050331000
	6	3.394138000	-3.122257000	0.098599000
	6	4.383524000	-2.708035000	-1.013469000
	6	5.405606000	-1.710548000	-0.473469000
	6	4.678412000	-0.454157000	0.049343000
	7	6.328614000	-1.360221000	-1.544828000
	1	6.725413000	-2.220839000	-1.910666000
	1	7.097789000	-0.812372000	-1.172382000
	8	5.092835000	-3.840457000	-1.487640000
	1	4.537410000	-4.305241000	-2.117560000
	6	2.050785000	-3.594671000	-0.439024000
	1	3.409952000	2.539235000	2.002441000
	1	2.002774000	1.995171000	2.902606000
	1	0.800083000	3.869017000	2.268421000
	1	2.242884000	4.494830000	1.508970000
	1	2.626852000	1.508586000	-0.014174000
	1	-5.975091000	0.770783000	-1.196885000
	1	-7.430531000	-1.199620000	-0.689786000
	1	-6.451724000	-3.251044000	0.218745000
	1	-5.382585000	-4.447636000	1.839289000
	1	-5.156852000	-5.127897000	0.198689000
	1	-3.957888000	-5.456204000	1.474414000
	1	0.983177000	6.166435000	-1.273038000
	1	-0.782853000	6.341168000	-1.230532000
	1	-0.016696000	4.958741000	-2.085767000

	1	2.579808000	-0.784760000	-0.424856000
	1	3.882495000	-3.927377000	0.656046000
	1	3.849919000	-2.226780000	-1.848117000
	1	5.898046000	-2.207062000	0.379329000
	1	4.673944000	0.302849000	-0.741459000
	1	5.190350000	-0.043953000	0.924345000
	1	1.401060000	-3.925140000	0.372247000
	1	1.523342000	-2.792970000	-0.964029000
	1	2.193702000	-4.428364000	-1.135358000
**Doxorubicin**	6	-7.520322000	-0.453390000	-0.034910000
	6	-7.279723000	0.889055000	-0.311964000
	6	-5.982610000	1.405058000	-0.240156000
	6	-4.913408000	0.545784000	0.097415000
	6	-5.182311000	-0.803150000	0.349779000
	6	-6.481179000	-1.304825000	0.300997000
	6	-3.515424000	1.040378000	0.264687000
	6	-2.431231000	0.014554000	0.089147000
	6	-2.689143000	-1.344356000	0.377171000
	6	-4.082843000	-1.764059000	0.697895000
	6	-1.150681000	0.401933000	-0.298511000
	6	-0.128380000	-0.556168000	-0.406662000
	6	-0.343129000	-1.874558000	-0.024866000
	6	-1.641978000	-2.276004000	0.344911000
	6	1.192356000	-0.099407000	-0.976069000
	6	2.150544000	-1.250235000	-1.247463000
	6	2.162547000	-2.243025000	-0.078021000
	6	0.778289000	-2.886891000	0.019759000
	8	-5.683284000	2.689568000	-0.509473000
	6	-6.724857000	3.566409000	-0.874339000
	8	-4.360351000	-2.847156000	1.176281000
	8	-3.261635000	2.188506000	0.563081000
	8	-1.892972000	-3.564621000	0.660185000
	8	-0.885848000	1.689033000	-0.638092000
	8	1.726488000	0.872072000	-0.040238000
	1	0.995752000	0.435646000	-1.916221000
	6	2.834791000	1.610125000	-0.465687000
	6	2.850105000	2.930851000	0.284617000
	6	4.111726000	3.721128000	-0.060925000
	6	5.355352000	2.840735000	0.170841000
	6	5.201241000	1.496785000	-0.542958000
	8	3.985024000	0.857319000	-0.167084000
	6	6.326665000	0.530063000	-0.232243000
	8	5.540919000	2.627642000	1.542350000
	7	4.222130000	4.880537000	0.821027000
	8	2.494643000	-1.636048000	1.153173000
	6	3.230679000	-3.298428000	-0.404033000
	6	4.574014000	-3.068029000	0.259055000
	8	5.543560000	-3.870236000	-0.353635000
	8	3.011405000	-4.204960000	-1.172727000
	1	-1.118516000	-4.099668000	0.465704000
	1	0.011353000	1.879827000	-0.334268000
	1	3.328087000	5.346117000	0.930596000

	1	4.874628000	5.560360000	0.444596000
	1	5.248749000	3.454116000	1.958422000
	1	6.337654000	-3.830599000	0.183575000
	1	-8.536478000	-0.830937000	-0.089663000
	1	-8.109945000	1.530106000	-0.581027000
	1	-6.644293000	-2.351802000	0.525958000
	1	3.151586000	-0.860409000	-1.440292000
	1	1.817886000	-1.782873000	-2.143513000
	1	0.690945000	-3.627329000	-0.786188000
	1	0.743508000	-3.424725000	0.975596000
	1	-7.218875000	3.247167000	-1.800605000
	1	-7.473788000	3.658443000	-0.078274000
	1	-6.252453000	4.535030000	-1.034304000
	1	2.778162000	1.777319000	-1.559114000
	1	2.836713000	2.709463000	1.355675000
	1	1.952808000	3.508207000	0.034717000
	1	4.076835000	3.991092000	-1.129859000
	1	6.231103000	3.348196000	-0.274533000
	1	5.172127000	1.702086000	-1.627817000
	1	6.340501000	0.321094000	0.839043000
	1	7.288042000	0.967096000	-0.514507000
	1	6.192217000	-0.403275000	-0.785044000
	1	2.548028000	-0.679143000	1.046195000
	1	4.435693000	-3.283940000	1.329554000
	1	4.796910000	-1.991375000	0.203034000
**Chlortetracycline**	6	2.763517000	-1.221950000	0.344543000
	8	3.172110000	-2.361474000	0.528986000
	6	3.091593000	-0.083566000	1.185734000
	8	3.737220000	0.840735000	3.258519000
	6	3.537435000	-0.191088000	2.583308000
	7	3.715543000	-1.419015000	3.092878000
	6	3.130964000	1.153777000	0.588314000
	8	3.395592000	2.255522000	1.233670000
	6	2.944023000	1.250853000	-0.909717000
	7	4.127161000	0.694659000	-1.566083000
	6	5.344251000	1.372843000	-1.140696000
	6	4.014478000	0.704857000	-3.017822000
	6	1.650185000	0.484396000	-1.243055000
	6	0.466914000	1.200160000	-0.586616000
	6	-0.857417000	0.508656000	-0.896275000
	6	-2.055221000	1.320999000	-0.327803000
	6	-2.344585000	2.410972000	-1.381256000
	6	-3.315859000	0.462655000	-0.078926000
	6	-4.536106000	1.032339000	0.281862000
	6	-5.703069000	0.275057000	0.405936000
	6	-5.676892000	-1.087079000	0.226887000
	6	-4.456065000	-1.725619000	-0.010846000
	8	-4.475392000	-3.054319000	-0.068345000
	6	-3.274959000	-0.953608000	-0.170308000
	6	-1.988846000	-1.664225000	-0.254419000
	8	-1.967991000	-2.920085000	-0.094235000
	6	-0.776489000	-0.929820000	-0.467832000

	6	0.419103000	-1.604499000	-0.451040000
	8	0.514141000	-2.891148000	-0.197512000
	6	1.775492000	-0.995283000	-0.836762000
	8	2.236612000	-1.738001000	-1.941133000
	1	-3.536320000	-3.351524000	-0.101679000
	1	-0.418750000	-3.225816000	-0.091190000
	1	2.599221000	-2.554634000	-1.568367000
	1	4.057644000	-1.494764000	4.036310000
	1	3.670616000	-2.220032000	2.475459000
	1	3.545530000	1.961811000	2.196863000
	1	2.801784000	2.318297000	-1.160694000
	1	5.498183000	1.253291000	-0.065841000
	1	5.336185000	2.454140000	-1.366492000
	1	6.198887000	0.920212000	-1.648548000
	1	4.963989000	0.370638000	-3.442387000
	1	3.794604000	1.709047000	-3.424440000
	1	3.248866000	0.001012000	-3.343881000
	1	1.491743000	0.490301000	-2.325136000
	1	0.582039000	1.238491000	0.499266000
	1	-0.998294000	0.509236000	-1.986886000
	1	-6.630884000	0.775249000	0.659458000
	1	-6.569698000	-1.692413000	0.325830000
	1	-1.408266000	2.888576000	-1.679124000
	1	-2.800724000	1.966096000	-2.270018000
	1	-3.008439000	3.188975000	-1.013178000
	1	0.454173000	2.237081000	-0.934221000
	17	-4.723230000	2.733520000	0.722612000
	8	-1.622281000	1.876882000	0.901679000
	1	-2.310856000	2.467510000	1.224609000
**Oxytetracycline**	6	1.952726000	-1.589238000	0.322585000
	8	1.583320000	-2.580842000	0.937514000
	6	3.283428000	-1.003987000	0.439178000
	8	5.562496000	-1.239980000	1.052412000
	6	4.406922000	-1.723831000	1.071798000
	7	4.160711000	-2.897626000	1.665739000
	6	3.542743000	0.260956000	-0.064083000
	8	4.740017000	0.773692000	-0.055978000
	6	2.464006000	1.196233000	-0.577043000
	7	2.544132000	2.540387000	-0.024666000
	6	3.388746000	3.463706000	-0.764545000
	6	2.762896000	2.619550000	1.409707000
	6	1.064832000	0.615866000	-0.343850000
	6	-0.013371000	1.404371000	-1.080507000
	8	0.164678000	1.356140000	-2.483577000
	6	-1.409527000	0.904329000	-0.678955000
	6	-2.033062000	1.436184000	0.629037000
	6	-1.872853000	2.943662000	0.817997000
	6	-3.486985000	0.955678000	0.658558000
	6	-4.504507000	1.737356000	1.178835000
	6	-5.821510000	1.262023000	1.216239000
	6	-6.145506000	0.001752000	0.761668000
	6	-5.136724000	-0.820700000	0.244641000

	8	-5.482384000	-2.044286000	-0.158165000
	6	-3.809085000	-0.337923000	0.160291000
	6	-2.770810000	-1.198257000	-0.411170000
	8	-2.981661000	-2.416731000	-0.648544000
	6	-1.468980000	-0.602570000	-0.635465000
	6	-0.380952000	-1.412066000	-0.726430000
	8	-0.444792000	-2.724650000	-0.907503000
	6	1.033026000	-0.874762000	-0.701757000
	8	1.587203000	-1.039389000	-2.012792000
	1	-4.658230000	-2.510433000	-0.418431000
	1	-1.405884000	-2.957609000	-0.931623000
	1	1.474821000	-1.966848000	-2.253505000
	1	4.932304000	-3.378609000	2.097216000
	1	3.207821000	-3.241050000	1.702471000
	1	5.338463000	0.050950000	0.370818000
	1	2.603583000	1.275832000	-1.660302000
	1	3.128917000	3.423931000	-1.825774000
	1	4.463320000	3.257246000	-0.654130000
	1	3.193730000	4.481184000	-0.410453000
	1	2.520837000	3.630290000	1.751401000
	1	3.800166000	2.396397000	1.705769000
	1	2.101325000	1.926432000	1.937866000
	1	0.835314000	0.676746000	0.728498000
	1	0.100000000	2.455298000	-0.812624000
	1	0.436691000	0.461857000	-2.725911000
	1	-2.061675000	1.238064000	-1.497788000
	1	-1.520302000	0.939229000	1.467303000
	1	-4.298156000	2.730130000	1.557282000
	1	-6.602590000	1.899214000	1.619725000
	1	-7.157685000	-0.383277000	0.800894000
	1	-0.815315000	3.216039000	0.852571000
	1	-2.344159000	3.501350000	0.002284000
	1	-2.310936000	3.276685000	1.761617000
**Esperamycin A1**	6	3.922075000	0.484235000	-0.067686000
	6	5.065484000	0.675778000	-1.058237000
	6	5.281361000	2.175671000	-1.238610000
	6	3.980735000	2.893774000	-1.614963000
	6	2.842199000	2.496079000	-0.661932000
	6	1.478290000	2.983994000	-1.106386000
	8	3.689102000	-0.868172000	0.091566000
	8	6.275497000	0.098305000	-0.601851000
	8	6.222086000	2.436196000	-2.261795000
	7	4.121472000	4.342700000	-1.687565000
	8	2.768004000	1.079966000	-0.601256000
	1	4.171309000	0.953578000	0.899800000
	1	4.758736000	0.226446000	-2.009973000
	1	5.634843000	2.583918000	-0.280686000
	1	3.704000000	2.579036000	-2.630077000
	1	3.070765000	2.891009000	0.340583000
	1	0.722267000	2.663985000	-0.384701000
	1	1.468584000	4.072109000	-1.189035000
	1	1.233388000	2.559743000	-2.085444000

	1	7.078504000	2.147799000	-1.933372000
	1	4.893260000	4.544196000	-2.321793000
	6	6.824618000	-0.888669000	-1.457912000
	6	8.221386000	-1.213912000	-0.942734000
	6	8.156545000	-2.005933000	0.362810000
	6	7.260081000	-3.233294000	0.206207000
	6	5.911338000	-2.770196000	-0.338198000
	6	6.368128000	-5.090980000	1.678460000
	6	6.528582000	-6.087542000	0.522867000
	6	4.892471000	-4.827622000	1.997417000
	6	10.222356000	-1.494111000	1.403879000
	8	9.434259000	-2.466820000	0.764778000
	7	7.120753000	-3.837314000	1.523940000
	8	6.030083000	-2.031126000	-1.549910000
	1	6.863017000	-0.493519000	-2.481280000
	1	8.783724000	-0.284695000	-0.813854000
	1	8.732810000	-1.820178000	-1.696764000
	1	7.722907000	-1.379539000	1.157126000
	1	7.733973000	-3.897688000	-0.538095000
	1	5.265310000	-3.612768000	-0.591427000
	1	5.415753000	-2.169115000	0.426195000
	1	6.796086000	-5.563580000	2.571432000
	1	7.584148000	-6.307738000	0.334653000
	1	6.019624000	-7.027300000	0.759344000
	1	6.093472000	-5.700171000	-0.404413000
	1	4.354572000	-4.388333000	1.153462000
	1	4.372628000	-5.760521000	2.236922000
	1	4.808583000	-4.148889000	2.851157000
	1	11.143204000	-1.985493000	1.723671000
	1	10.487061000	-0.663393000	0.734601000
	1	9.713485000	-1.079089000	2.286096000
	1	8.063500000	-3.964691000	1.874431000
	6	-7.186022000	-0.287953000	-0.204153000
	6	-8.581244000	-0.147812000	0.249486000
	6	-9.230977000	-1.262079000	0.821466000
	6	-10.535072000	-1.198856000	1.256128000
	6	-11.236497000	0.024817000	1.120061000
	6	-10.615273000	1.130670000	0.564595000
	6	-9.284217000	1.065652000	0.123528000
	6	-9.168780000	3.421904000	-0.654306000
	6	-8.181208000	4.375413000	-1.285502000
	6	-8.543452000	5.628481000	-1.563449000
	6	-10.562134000	-3.448807000	1.968343000
	6	-13.255243000	1.210576000	1.451083000
	6	-5.973265000	4.599493000	-2.096274000
	7	-8.651691000	2.178248000	-0.435320000
	8	-6.499680000	0.603218000	-0.680249000
	8	-11.230520000	-2.218455000	1.816386000
	8	-12.506214000	0.016154000	1.562324000
	8	-10.304741000	3.786977000	-0.389655000
	8	-6.974493000	3.802221000	-1.505093000
	1	-8.666267000	-2.178935000	0.915354000
	1	-11.135730000	2.069192000	0.455187000

	1	-9.554815000	5.924950000	-1.321135000
	1	-7.878388000	6.352252000	-2.015646000
	1	-9.682948000	-3.354292000	2.618741000
	1	-11.278969000	-4.127473000	2.430196000
	1	-10.247287000	-3.860698000	1.000658000
	1	-12.798888000	2.024330000	2.026145000
	1	-13.356725000	1.523635000	0.405710000
	1	-14.239094000	0.982996000	1.860173000
	1	-5.092581000	3.965023000	-2.187815000
	1	-6.284376000	4.947585000	-3.088740000
	1	-5.741169000	5.467774000	-1.467554000
	1	-7.681314000	2.024469000	-0.707806000
	6	3.930257000	5.936590000	-0.031264000
	6	4.208101000	6.164269000	1.444286000
	6	3.589568000	7.484042000	1.895172000
	6	4.003000000	8.626021000	0.946128000
	6	3.772773000	8.237314000	-0.525525000
	6	4.318242000	9.249307000	-1.515323000
	6	4.444304000	11.348052000	1.494010000
	8	4.603077000	4.783335000	-0.414652000
	8	2.184796000	7.300045000	1.898160000
	8	4.438673000	7.012177000	-0.788691000
	16	3.087955000	10.147750000	1.405369000
	1	2.852840000	5.830114000	-0.226213000
	1	5.293243000	6.190882000	1.584464000
	1	3.795200000	5.348003000	2.042494000
	1	3.944397000	7.727603000	2.906479000
	1	5.071818000	8.824306000	1.089042000
	1	2.689378000	8.109579000	-0.678589000
	1	4.163738000	8.885923000	-2.533442000
	1	5.393181000	9.387332000	-1.361000000
	1	3.811269000	10.210609000	-1.405273000
	1	4.002923000	12.308521000	1.765573000
	1	5.173085000	11.066108000	2.256649000
	1	4.938748000	11.448722000	0.525584000
	1	1.790205000	8.068446000	2.324670000
	6	-3.466979000	-3.361546000	-0.119749000
	6	-4.855312000	-2.969792000	0.368241000
	6	-5.354307000	-1.794239000	-0.450785000
	6	-5.325473000	-2.139072000	-1.941170000
	6	-3.908248000	-2.566105000	-2.325904000
	6	-3.810382000	-3.066396000	-3.753310000
	8	-2.615070000	-2.279371000	0.225638000
	8	-6.715700000	-1.540032000	-0.048637000
	8	-6.223892000	-3.188882000	-2.230512000
	8	-3.438939000	-3.628590000	-1.489684000
	1	-3.116612000	-4.276475000	0.364002000
	1	-5.522335000	-3.822943000	0.222710000
	1	-4.813630000	-2.721131000	1.430467000
	1	-4.754234000	-0.901460000	-0.267117000
	1	-5.575874000	-1.236457000	-2.518855000
	1	-3.256257000	-1.694925000	-2.187572000
	1	-2.778299000	-3.346304000	-3.976204000

	1 -4.456765000 -3.934530000 -3.892536000<br>1 -4.119163000 -2.281875000 -4.450131000<br>1 -7.075612000 -2.938016000 -1.859463000<br>6 2.830506000 -1.312473000 1.121467000<br>6 2.394430000 -0.261180000 2.053889000<br>6 1.800105000 0.568841000 2.704167000<br>6 0.844008000 1.487546000 3.224087000<br>6 -0.380054000 1.538753000 2.651178000<br>6 -0.689370000 0.674856000 1.562609000<br>6 -0.750101000 -0.130289000 0.661723000<br>6 -0.618983000 -1.116423000 -0.428973000<br>6 -1.265887000 -2.474147000 -0.074927000<br>6 -0.551516000 -3.150170000 1.110414000<br>6 0.875098000 -2.799496000 1.370690000<br>6 1.533912000 -1.928642000 0.583389000<br>6 0.869641000 -1.451259000 -0.646288000<br>6 1.356985000 -1.477685000 -1.887954000<br>6 2.658223000 -2.040481000 -2.345578000<br>6 3.823640000 -6.691286000 -2.300449000<br>6 1.547683000 -4.548114000 2.894036000<br>6 1.975172000 -6.055725000 4.619088000<br>8 -1.248879000 -0.652140000 -1.597954000<br>8 -1.170142000 -3.862203000 1.870622000<br>8 1.541741000 -5.439941000 2.078639000<br>8 1.748387000 -4.708538000 4.215996000<br>7 1.413316000 -3.210679000 2.609702000<br>16 2.399668000 -3.435825000 -3.510203000<br>16 1.438384000 -4.840352000 -2.328702000<br>16 2.893939000 -5.676020000 -1.115047000<br>1 3.370789000 -2.096150000 1.665860000<br>1 1.092988000 2.131300000 4.060540000<br>1 -1.131548000 2.229658000 3.019482000<br>1 -1.143014000 -3.103718000 -0.968843000<br>1 0.686620000 -1.150793000 -2.678026000<br>1 3.304617000 -2.374078000 -1.537816000<br>1 3.225968000 -1.313554000 -2.935389000<br>1 4.615930000 -7.182812000 -1.730036000<br>1 4.274659000 -6.062832000 -3.071460000<br>1 3.179632000 -7.445834000 -2.754226000<br>1 2.044344000 -6.029847000 5.705903000<br>1 1.150985000 -6.697722000 4.302278000<br>1 2.906382000 -6.434877000 4.190366000<br>1 -1.014273000 0.275296000 -1.706252000<br>1 1.320335000 -2.585186000 3.398327000
**Q2K@Dicentrine**	7 5.923322000 1.697660000 -2.143412000<br>6 5.837720000 0.383356000 -2.393883000<br>6 4.704601000 -0.385753000 -2.605430000<br>6 3.432803000 0.246165000 -2.535766000<br>7 3.548947000 1.611913000 -2.282494000<br>6 4.734999000 2.282006000 -2.102543000<br>7 6.878331000 -0.503101000 -2.470945000<br>6 6.337351000 -1.742344000 -2.715451000

	7	5.036770000	-1.711272000	-2.802097000
	7	4.665521000	3.602419000	-1.884232000
	7	1.228463000	-2.857688000	-3.099002000
	6	-0.156025000	-2.759846000	-2.983186000
	6	-0.787012000	-4.027690000	-3.098215000
	6	0.005233000	-5.144820000	-3.307489000
	7	1.337263000	-5.211986000	-3.439364000
	6	1.917413000	-4.027250000	-3.315850000
	7	-2.118599000	-4.375397000	-2.998354000
	6	-2.132893000	-5.671121000	-3.142065000
	7	-0.875758000	-6.193251000	-3.335050000
	8	-0.696868000	-1.663592000	-2.798392000
	7	3.251172000	-3.940437000	-3.415779000
	7	-3.267808000	-0.523120000	-2.817407000
	6	-4.457879000	-1.206489000	-2.898893000
	7	-5.645119000	-0.617091000	-2.897041000
	6	-5.552671000	0.716750000	-2.820974000
	6	-4.413976000	1.503755000	-2.749660000
	6	-3.146520000	0.861039000	-2.722373000
	7	-4.744072000	2.840736000	-2.647195000
	6	-6.047834000	2.861808000	-2.663161000
	7	-6.592894000	1.605517000	-2.769864000
	7	-4.393651000	-2.540004000	-2.997860000
	8	-2.032909000	1.390303000	-2.613829000
	7	-0.945747000	3.975218000	-2.265277000
	6	0.428425000	3.840890000	-2.082224000
	6	1.050759000	5.089816000	-1.811275000
	6	0.260430000	6.227180000	-1.767277000
	7	-1.060247000	6.335376000	-1.965397000
	6	-1.632664000	5.164456000	-2.207443000
	7	2.370537000	5.392313000	-1.542294000
	6	2.378634000	6.680023000	-1.337469000
	7	1.129412000	7.240698000	-1.460344000
	8	0.968536000	2.731246000	-2.154832000
	7	-2.955251000	5.117306000	-2.412666000
	1	-1.455289000	3.094433000	-2.443955000
	1	-3.502618000	4.255042000	-2.519288000
	1	-3.433934000	5.999282000	-2.356574000
	1	-2.379490000	-1.048897000	-2.811978000
	1	-5.278610000	-3.016717000	-3.003704000
	1	-3.532502000	-3.093101000	-2.941900000
	1	-0.635721000	-7.157433000	-3.497968000
	1	1.743667000	-1.969500000	-2.995977000
	1	3.732645000	-4.815777000	-3.527320000
	1	3.795109000	-3.098564000	-3.197936000
	1	2.657959000	2.129096000	-2.222108000
	1	5.545047000	4.061745000	-1.724134000
	1	3.797455000	4.131423000	-1.755528000
	1	6.953055000	-2.623681000	-2.814952000
	1	3.247674000	7.271385000	-1.090690000
	1	-6.663047000	3.746873000	-2.600221000
	1	-3.012648000	-6.296483000	-3.112824000
	1	7.854407000	-0.270695000	-2.387007000

	1	0.889182000	8.215204000	-1.379114000
	1	-7.569912000	1.367240000	-2.818865000
	19	0.086186000	0.350604000	-1.157343000
	7	2.957413000	5.045072000	1.764796000
	6	3.899661000	4.102760000	1.607003000
	6	3.747831000	2.787525000	1.205284000
	6	2.441864000	2.318698000	0.892187000
	7	1.486932000	3.319726000	1.040139000
	6	1.748598000	4.600065000	1.460972000
	7	5.246165000	4.252739000	1.810527000
	6	5.834476000	3.043423000	1.529880000
	7	4.964666000	2.142113000	1.164735000
	8	2.115621000	1.192233000	0.502277000
	7	0.700834000	5.429005000	1.582223000
	7	3.305367000	-1.339292000	0.295752000
	6	2.287866000	-2.257915000	0.058000000
	6	2.789340000	-3.581504000	-0.069136000
	6	4.149655000	-3.791365000	0.081467000
	7	5.108770000	-2.893222000	0.343501000
	6	4.631435000	-1.661493000	0.439784000
	7	2.131414000	-4.769878000	-0.302124000
	6	3.071077000	-5.674590000	-0.304889000
	7	4.315852000	-5.138934000	-0.085989000
	8	1.111552000	-1.887264000	-0.026255000
	7	5.484698000	-0.657661000	0.686554000
	7	-1.436769000	-3.061656000	0.087452000
	6	-1.728545000	-4.402754000	0.137879000
	7	-2.950100000	-4.888214000	0.287450000
	6	-3.877219000	-3.922543000	0.350804000
	6	-3.701379000	-2.552702000	0.257567000
	6	-2.382626000	-2.042926000	0.133022000
	7	-4.907418000	-1.895417000	0.366733000
	6	-5.789785000	-2.841373000	0.522562000
	7	-5.226001000	-4.092506000	0.502600000
	7	-0.705175000	-5.260090000	0.006961000
	8	-2.037151000	-0.854824000	0.066097000
	7	-3.257381000	1.638694000	0.419893000
	6	-2.245985000	2.587731000	0.523025000
	6	-2.760759000	3.893427000	0.742824000
	6	-4.135098000	4.065534000	0.780204000
	7	-5.094180000	3.135153000	0.687321000
	6	-4.601369000	1.914947000	0.503784000
	7	-2.106663000	5.098940000	0.890736000
	6	-3.059602000	5.980741000	1.005885000
	7	-4.311113000	5.413357000	0.945742000
	8	-1.057267000	2.260674000	0.421875000
	7	-5.446275000	0.886493000	0.391982000
	1	-2.937443000	0.659243000	0.305302000
	1	-5.164909000	-0.107585000	0.378357000
	1	-6.417808000	1.114024000	0.515336000
	1	-0.453293000	-2.753984000	0.040947000
	1	-0.953753000	-6.233540000	0.008789000
	1	0.266527000	-4.983420000	-0.163445000

	1	5.184571000	-5.638703000	0.010409000
	1	2.993249000	-0.364623000	0.432924000
	1	6.449243000	-0.921517000	0.786056000
	1	5.220565000	0.330089000	0.773617000
	1	0.516368000	3.043743000	0.829086000
	1	0.926058000	6.371782000	1.848785000
	1	-0.249496000	5.220886000	1.258205000
	1	6.900493000	2.892026000	1.612119000
	1	-2.919794000	7.043978000	1.132145000
	1	-6.851677000	-2.694458000	0.650697000
	1	2.924174000	-6.731351000	-0.471762000
	1	5.701380000	5.091702000	2.131662000
	1	-5.197221000	5.883745000	1.033176000
	1	-5.688309000	-4.969856000	0.674801000
	8	2.319976000	-0.278683000	-2.658746000
	6	-3.246583000	-2.021091000	3.407971000
	6	-3.599435000	-0.661946000	3.381500000
	6	-2.588680000	0.291744000	3.306445000
	6	-1.248143000	-0.083850000	3.255402000
	6	-0.893146000	-1.434688000	3.325852000
	6	-1.913432000	-2.392239000	3.397085000
	6	-0.159943000	0.939399000	3.098812000
	6	1.030086000	0.603061000	3.992780000
	6	1.525793000	-0.793043000	3.633391000
	6	0.542543000	-1.781244000	3.344649000
	7	2.080368000	1.617122000	3.892777000
	6	3.288309000	1.186258000	4.583297000
	6	3.898928000	-0.016427000	3.889576000
	6	2.882930000	-1.103166000	3.649816000
	6	3.332333000	-2.414807000	3.412128000
	6	2.378075000	-3.364858000	3.156434000
	6	1.026938000	-3.053008000	3.105867000
	8	2.557927000	-4.691076000	2.864199000
	6	1.250943000	-5.248283000	2.862787000
	8	0.325850000	-4.179783000	2.749962000
	1	0.664332000	0.554496000	5.042922000
	8	-4.927503000	-0.375383000	3.432518000
	6	-5.297389000	0.935489000	3.806888000
	8	-4.210872000	-2.997150000	3.409908000
	6	-4.877547000	-3.157860000	4.651598000
	6	1.649315000	2.903634000	4.415548000
	1	-2.836173000	1.347324000	3.269744000
	1	-1.687969000	-3.450382000	3.434849000
	1	0.185527000	0.946837000	2.060224000
	1	-0.560494000	1.931248000	3.318272000
	1	4.002721000	2.016113000	4.572339000
	1	3.079390000	0.949747000	5.645340000
	1	4.315769000	0.305973000	2.929271000
	1	4.732259000	-0.404111000	4.485557000
	1	4.388694000	-2.659791000	3.417815000
	1	1.137137000	-5.907499000	2.001105000
	1	1.080839000	-5.783793000	3.808131000
	1	-4.806875000	1.234003000	4.741299000

	1	-5.067417000	1.671766000	3.029079000
	1	-6.377495000	0.913687000	3.955911000
	1	-4.165132000	-3.405836000	5.448107000
	1	-5.431522000	-2.254627000	4.926357000
	1	-5.576141000	-3.986997000	4.525748000
	1	1.343180000	2.852794000	5.477155000
	1	2.472877000	3.618152000	4.329815000
	1	0.815129000	3.307154000	3.839391000
**Q2K@Chlortetracycline**	7	-1.390871000	-5.492407000	-0.647345000
	6	-0.051064000	-5.467888000	-0.626367000
	6	0.798150000	-4.372867000	-0.603063000
	6	0.221543000	-3.073932000	-0.573937000
	7	-1.168689000	-3.126644000	-0.594486000
	6	-1.916810000	-4.277618000	-0.638035000
	7	0.781575000	-6.555262000	-0.615733000
	6	2.068466000	-6.078751000	-0.570377000
	7	2.117363000	-4.774651000	-0.561091000
	7	-3.248143000	-4.139070000	-0.686495000
	7	3.484094000	-1.084363000	-0.473049000
	6	3.431801000	0.291247000	-0.332704000
	6	4.703377000	0.899309000	-0.484491000
	6	5.800311000	0.081969000	-0.693789000
	7	5.839576000	-1.255467000	-0.781300000
	6	4.638628000	-1.805732000	-0.652675000
	7	5.078207000	2.222102000	-0.383519000
	6	6.371407000	2.212066000	-0.554276000
	7	6.865083000	0.943102000	-0.750935000
	8	2.349284000	0.858548000	-0.117226000
	7	4.517969000	-3.135802000	-0.707535000
	7	1.416115000	3.183647000	0.988388000
	6	2.190073000	4.239489000	1.402363000
	7	1.706032000	5.321095000	1.990934000
	6	0.371021000	5.282908000	2.108686000
	6	-0.505131000	4.287167000	1.703866000
	6	0.030763000	3.128314000	1.082942000
	7	-1.810741000	4.632825000	1.985784000
	6	-1.727841000	5.806766000	2.547136000
	7	-0.431649000	6.252287000	2.647695000
	7	3.515855000	4.132741000	1.208020000
	8	-0.590506000	2.142015000	0.659636000
	7	-3.262293000	1.356318000	0.402563000
	6	-3.229320000	0.040975000	-0.044525000
	6	-4.526905000	-0.471244000	-0.289722000
	6	-5.615776000	0.357885000	-0.075351000
	7	-5.627037000	1.623029000	0.369960000
	6	-4.409477000	2.089265000	0.599135000
	7	-4.926710000	-1.724718000	-0.698669000
	6	-6.227112000	-1.655364000	-0.744687000
	7	-6.701673000	-0.414531000	-0.385323000
	8	-2.147586000	-0.548327000	-0.177401000
	7	-4.254500000	3.349396000	1.027511000
	1	-2.338577000	1.788698000	0.565556000

	1	-3.364309000	3.730227000	1.364912000
	1	-5.104975000	3.832022000	1.261508000
	1	1.869884000	2.360913000	0.551238000
	1	4.052108000	4.944080000	1.465149000
	1	3.945716000	3.451850000	0.572593000
	1	7.829506000	0.677627000	-0.867342000
	1	2.585278000	-1.580152000	-0.368888000
	1	5.366980000	-3.649681000	-0.864408000
	1	3.628796000	-3.639112000	-0.617446000
	1	-1.645722000	-2.216390000	-0.510400000
	1	-3.783448000	-4.986440000	-0.611881000
	1	-3.744230000	-3.243521000	-0.677294000
	1	2.921968000	-6.740329000	-0.546119000
	1	-6.887578000	-2.457603000	-1.038085000
	1	-2.561955000	6.391057000	2.906927000
	1	7.010264000	3.082731000	-0.557400000
	1	0.487107000	-7.517990000	-0.586760000
	1	-7.664604000	-0.129458000	-0.313022000
	1	-0.113630000	7.110896000	3.067547000
	19	0.033157000	0.464656000	-1.471460000
	7	-4.833596000	-2.435712000	-3.936752000
	6	-3.812476000	-3.304419000	-3.935317000
	6	-2.463968000	-3.051536000	-3.740814000
	6	-2.059611000	-1.718531000	-3.469265000
	7	-3.137587000	-0.838893000	-3.449222000
	6	-4.444032000	-1.195395000	-3.683085000
	7	-3.886974000	-4.658921000	-4.116913000
	6	-2.607655000	-5.152067000	-4.019319000
	7	-1.725952000	-4.217088000	-3.797170000
	8	-0.912129000	-1.308329000	-3.255743000
	7	-5.358228000	-0.216020000	-3.656113000
	7	1.719571000	-2.163293000	-3.607090000
	6	2.542912000	-1.040625000	-3.579281000
	6	3.903926000	-1.377729000	-3.816351000
	6	4.231288000	-2.715652000	-3.977123000
	7	3.420655000	-3.783641000	-3.979216000
	6	2.149359000	-3.453171000	-3.807667000
	7	5.030337000	-0.581305000	-3.885995000
	6	6.012378000	-1.416824000	-4.078540000
	7	5.590280000	-2.722340000	-4.139825000
	8	2.065277000	0.078951000	-3.353965000
	7	1.213985000	-4.411722000	-3.831830000
	7	2.991725000	2.692354000	-2.888723000
	6	4.263158000	3.164691000	-3.110438000
	7	4.662316000	4.386760000	-2.787499000
	6	3.691745000	5.105079000	-2.207478000
	6	2.380251000	4.737017000	-1.952284000
	6	1.963113000	3.427646000	-2.311709000
	7	1.688956000	5.756921000	-1.329127000
	6	2.562647000	6.717427000	-1.205150000
	7	3.793167000	6.380750000	-1.717756000
	7	5.127055000	2.330759000	-3.707165000
	8	0.840800000	2.930771000	-2.155972000

	7	-1.787354000	3.809852000	-1.886587000
	6	-2.633499000	2.803544000	-2.350131000
	6	-3.988009000	3.232609000	-2.395611000
	6	-4.287623000	4.515036000	-1.961614000
	7	-3.454236000	5.464810000	-1.512959000
	6	-2.191770000	5.066461000	-1.504713000
	7	-5.133432000	2.557308000	-2.768847000
	6	-6.099068000	3.408951000	-2.564380000
	7	-5.647372000	4.611103000	-2.076088000
	8	-2.178278000	1.691498000	-2.645578000
	7	-1.240020000	5.918459000	-1.095322000
	1	-0.780492000	3.579682000	-1.893279000
	1	-0.233068000	5.740513000	-1.155309000
	1	-1.555969000	6.849372000	-0.884982000
	1	2.755265000	1.718860000	-3.142772000
	1	6.062414000	2.687889000	-3.802864000
	1	4.985316000	1.316395000	-3.776523000
	1	6.157812000	-3.538296000	-4.301061000
	1	0.712468000	-1.976630000	-3.474697000
	1	1.551269000	-5.346512000	-3.981997000
	1	0.205021000	-4.249941000	-3.757609000
	1	-2.904347000	0.141410000	-3.226290000
	1	-6.307344000	-0.505236000	-3.816403000
	1	-5.172382000	0.749831000	-3.365688000
	1	-2.395188000	-6.205986000	-4.118876000
	1	-7.147685000	3.227516000	-2.747111000
	1	2.379051000	7.679598000	-0.750785000
	1	7.053860000	-1.151703000	-4.182498000
	1	-4.727957000	-5.181853000	-4.299382000
	1	-6.198591000	5.424565000	-1.856059000
	1	4.612317000	6.964779000	-1.765957000
	8	0.805183000	-1.983544000	-0.524375000
	6	2.121370000	-2.684281000	4.296960000
	8	1.928687000	-3.860590000	4.591539000
	6	2.797137000	-1.746517000	5.176010000
	8	3.731865000	-1.249352000	7.299253000
	6	3.167902000	-2.095909000	6.565206000
	7	2.891963000	-3.321127000	7.020507000
	6	3.160935000	-0.486017000	4.729347000
	8	3.769732000	0.371633000	5.495942000
	6	2.981753000	-0.042382000	3.297062000
	7	4.243177000	-0.120577000	2.548922000
	6	5.039629000	-1.330579000	2.724079000
	6	5.073698000	1.059981000	2.757480000
	6	1.729056000	-0.702574000	2.682885000
	6	0.522978000	0.008439000	3.322995000
	6	-0.853253000	-0.470849000	2.861026000
	6	-1.982349000	0.035149000	3.795779000
	6	-1.868480000	1.557424000	3.917074000
	6	-3.361621000	-0.500471000	3.306936000
	6	-4.595900000	0.130415000	3.463224000
	6	-5.800039000	-0.496330000	3.106865000
	6	-5.823182000	-1.777556000	2.625803000

	6	-4.619358000	-2.472819000	2.475448000
	8	-4.708093000	-3.724522000	2.031179000
	6	-3.396489000	-1.833838000	2.790842000
	6	-2.159802000	-2.609711000	2.616168000
	8	-2.238680000	-3.851678000	2.365274000
	6	-0.888982000	-1.966246000	2.736920000
	6	0.241543000	-2.732523000	2.667893000
	8	0.223161000	-4.025442000	2.407667000
	6	1.655726000	-2.215869000	2.878288000
	8	2.499762000	-2.850378000	1.933223000
	1	-3.794301000	-4.098758000	2.062627000
	1	-0.739877000	-4.281383000	2.323646000
	1	2.272704000	-3.789973000	1.937223000
	1	3.152849000	-3.541200000	7.967493000
	1	2.470353000	-3.993328000	6.389334000
	1	2.754096000	1.026758000	3.348125000
	1	4.433665000	-2.212641000	2.525686000
	1	5.491124000	-1.412636000	3.727629000
	1	5.851871000	-1.310018000	1.991601000
	1	5.932796000	1.004243000	2.081977000
	1	5.451864000	1.151855000	3.786928000
	1	4.512151000	1.965056000	2.508119000
	1	1.720459000	-0.509089000	1.608442000
	1	0.571615000	-0.084750000	4.414047000
	1	-1.049264000	-0.050229000	1.866981000
	1	-6.725531000	0.051632000	3.239572000
	1	-6.750260000	-2.278888000	2.375359000
	1	-0.842378000	1.817970000	4.171192000
	1	-2.129250000	2.062927000	2.985005000
	1	-2.497014000	1.949081000	4.711426000
	1	0.631849000	1.066415000	3.075360000
	17	-4.867540000	1.729088000	4.158801000
	8	-1.732324000	-0.568521000	5.061641000
	1	-2.372852000	-0.220458000	5.689493000
	1	3.877275000	-0.116009000	6.406734000
**Q2K@Oxytetracycline**	7	1.228328000	5.398109000	-0.937703000
	6	-0.100032000	5.263608000	-0.983423000
	6	-0.852684000	4.099700000	-0.972039000
	6	-0.174900000	2.859006000	-0.895410000
	7	1.199938000	3.021979000	-0.803314000
	6	1.850752000	4.234029000	-0.812572000
	7	-1.024215000	6.274287000	-1.029239000
	6	-2.264478000	5.691554000	-1.017338000
	7	-2.203377000	4.387263000	-0.988317000
	7	3.182517000	4.217955000	-0.689702000
	7	-3.363976000	0.539106000	-1.085200000
	6	-3.281507000	-0.836990000	-0.879337000
	6	-4.569727000	-1.419762000	-0.730344000
	6	-5.688058000	-0.611965000	-0.843320000
	7	-5.738874000	0.706875000	-1.082484000
	6	-4.536687000	1.246198000	-1.193867000
	7	-4.924714000	-2.727322000	-0.475540000

	6	-6.228352000	-2.712289000	-0.436023000
	7	-6.747479000	-1.458826000	-0.659737000
	8	-2.195328000	-1.418999000	-0.843446000
	7	-4.440394000	2.558393000	-1.470417000
	7	-1.065573000	-3.311710000	1.004334000
	6	-1.809226000	-4.416075000	1.352301000
	7	-1.355881000	-5.397667000	2.118781000
	6	-0.093281000	-5.197398000	2.517997000
	6	0.740148000	-4.127745000	2.236354000
	6	0.258712000	-3.098635000	1.384667000
	7	1.987620000	-4.312708000	2.797279000
	6	1.908130000	-5.461565000	3.408950000
	7	0.670188000	-6.043523000	3.276421000
	7	-3.061600000	-4.497720000	0.886351000
	8	0.888009000	-2.116511000	0.973309000
	7	3.400340000	-0.965076000	1.277981000
	6	3.371444000	0.239033000	0.586882000
	6	4.630254000	0.887441000	0.597402000
	6	5.690646000	0.266695000	1.236974000
	7	5.692943000	-0.888513000	1.916453000
	6	4.505195000	-1.473388000	1.917452000
	7	5.016318000	2.091264000	0.049730000
	6	6.281812000	2.195818000	0.341437000
	7	6.748754000	1.114153000	1.051214000
	8	2.323614000	0.630219000	0.051141000
	7	4.343933000	-2.626057000	2.582368000
	1	2.514219000	-1.497233000	1.269150000
	1	3.475008000	-3.172648000	2.598427000
	1	5.173228000	-3.004191000	3.005864000
	1	-1.491262000	-2.587783000	0.407994000
	1	-3.576973000	-5.311925000	1.172272000
	1	-3.532840000	-3.814375000	0.289769000
	1	-7.718434000	-1.190918000	-0.648382000
	1	-2.474153000	1.048880000	-1.107237000
	1	-5.308485000	3.061939000	-1.393798000
	1	-3.575816000	3.091081000	-1.353353000
	1	1.739084000	2.161802000	-0.613246000
	1	3.614142000	5.116729000	-0.557960000
	1	3.741593000	3.383136000	-0.483150000
	1	-3.172310000	6.276306000	-1.029479000
	1	6.923819000	3.018283000	0.064501000
	1	2.703980000	-5.933272000	3.965947000
	1	-6.862203000	-3.566580000	-0.249823000
	1	-0.815965000	7.259863000	-1.010888000
	1	7.675444000	0.987409000	1.423814000
	1	0.363062000	-6.918421000	3.669472000
	19	0.428143000	-0.789954000	-1.384963000
	7	5.822687000	1.847172000	-3.085814000
	6	4.856625000	2.692892000	-3.469830000
	6	3.487804000	2.479733000	-3.511236000
	6	2.986307000	1.216429000	-3.097859000
	7	4.005465000	0.360873000	-2.689927000
	6	5.341962000	0.680513000	-2.683137000

	7	5.019099000	3.982457000	-3.900062000
	6	3.767368000	4.478593000	-4.175850000
	7	2.824604000	3.604970000	-3.956831000
	8	1.805567000	0.848493000	-3.068744000
	7	6.192340000	-0.259409000	-2.245355000
	7	-0.682398000	1.725709000	-4.002136000
	6	-1.567428000	0.649047000	-3.961972000
	6	-2.900194000	1.052576000	-4.255092000
	6	-3.145079000	2.393743000	-4.503804000
	7	-2.275914000	3.416777000	-4.511586000
	6	-1.037675000	3.029704000	-4.247048000
	7	-4.068924000	0.318852000	-4.305674000
	6	-4.994376000	1.194597000	-4.588012000
	7	-4.492087000	2.464197000	-4.731854000
	8	-1.160479000	-0.483477000	-3.680728000
	7	-0.061927000	3.949031000	-4.189806000
	7	-2.194766000	-3.076052000	-3.175573000
	6	-3.484763000	-3.474543000	-3.421849000
	7	-3.969089000	-4.657171000	-3.071625000
	6	-3.072627000	-5.400142000	-2.409994000
	6	-1.764700000	-5.084170000	-2.077032000
	6	-1.259583000	-3.811526000	-2.457255000
	7	-1.176916000	-6.104892000	-1.357708000
	6	-2.103764000	-7.017268000	-1.260111000
	7	-3.273851000	-6.646768000	-1.878575000
	7	-4.278303000	-2.612065000	-4.077505000
	8	-0.140844000	-3.341421000	-2.211652000
	7	2.267540000	-4.140981000	-1.070919000
	6	3.190238000	-3.118912000	-1.284644000
	6	4.473705000	-3.451386000	-0.770401000
	6	4.630779000	-4.663616000	-0.117083000
	7	3.719912000	-5.623541000	0.096001000
	6	2.535053000	-5.317034000	-0.412649000
	7	5.655233000	-2.737641000	-0.779474000
	6	6.505037000	-3.497369000	-0.146535000
	7	5.941290000	-4.675958000	0.280552000
	8	2.851298000	-2.073642000	-1.852201000
	7	1.523603000	-6.185355000	-0.274962000
	1	1.322321000	-3.971012000	-1.456398000
	1	0.590409000	-6.069436000	-0.687646000
	1	1.758826000	-7.058112000	0.164906000
	1	-1.902984000	-2.120078000	-3.431087000
	1	-5.236204000	-2.907560000	-4.163531000
	1	-4.081591000	-1.609494000	-4.156971000
	1	-5.010181000	3.304668000	-4.927729000
	1	0.292467000	1.504102000	-3.743180000
	1	-0.338238000	4.887660000	-4.421123000
	1	0.937502000	3.734019000	-4.101831000
	1	3.699940000	-0.572681000	-2.372072000
	1	7.159746000	0.012787000	-2.220547000
	1	5.906578000	-1.116689000	-1.761206000
	1	3.619409000	5.486099000	-4.535588000
	1	7.540489000	-3.259001000	0.044976000

	1	-1.998642000	-7.968001000	-0.758784000
	1	-6.046667000	0.981950000	-4.703321000
	1	5.900403000	4.456188000	-4.014139000
	1	6.402695000	-5.434477000	0.755599000
	1	-4.117194000	-7.190529000	-1.963784000
	8	-0.681544000	1.721781000	-0.900680000
	6	-3.274031000	3.333836000	3.449930000
	8	-3.026566000	4.437952000	3.916523000
	6	-4.546144000	2.638954000	3.629512000
	8	-6.860060000	2.728772000	4.138565000
	6	-5.757616000	3.324858000	4.116890000
	7	-5.650963000	4.592934000	4.532551000
	6	-4.659996000	1.294497000	3.316016000
	8	-5.801325000	0.666293000	3.370673000
	6	-3.476359000	0.418850000	2.957728000
	7	-3.452700000	-0.861480000	3.644510000
	6	-4.146301000	-1.937025000	2.960985000
	6	-3.731646000	-0.823726000	5.068812000
	6	-2.149047000	1.162677000	3.150986000
	6	-0.988992000	0.348060000	2.579068000
	8	-1.231800000	0.007688000	1.235655000
	6	0.366839000	1.051378000	2.735732000
	6	1.205730000	0.743075000	3.994026000
	6	1.282531000	-0.749600000	4.319887000
	6	2.580459000	1.392036000	3.804408000
	6	3.734709000	0.815501000	4.309970000
	6	4.971635000	1.455478000	4.175288000
	6	5.086027000	2.677177000	3.546321000
	6	3.942686000	3.283102000	3.015383000
	8	4.091010000	4.467453000	2.415624000
	6	2.687606000	2.636637000	3.125201000
	6	1.496960000	3.293734000	2.587896000
	8	1.546897000	4.490071000	2.183419000
	6	0.257418000	2.551200000	2.579238000
	6	-0.914015000	3.235684000	2.438449000
	8	-0.980511000	4.516929000	2.108239000
	6	-2.265772000	2.569329000	2.552631000
	8	-2.815517000	2.472430000	1.242448000
	1	3.187038000	4.790564000	2.199305000
	1	-0.043829000	4.828909000	2.011261000
	1	-2.754413000	3.339000000	0.813214000
	1	-6.476872000	5.042225000	4.892149000
	1	-4.734348000	5.025017000	4.558462000
	1	-6.482136000	1.380622000	3.671085000
	1	-3.557789000	0.214602000	1.885385000
	1	-3.843105000	-1.951890000	1.911709000
	1	-5.243085000	-1.849605000	3.005835000
	1	-3.855233000	-2.893849000	3.407081000
	1	-3.396876000	-1.760498000	5.524656000
	1	-4.800131000	-0.690499000	5.300211000
	1	-3.176985000	-0.008894000	5.543734000
	1	-1.960015000	1.296665000	4.225529000
	1	-0.985556000	-0.606038000	3.104946000

	1	-1.149063000	0.796106000	0.678048000
	1	0.949910000	0.679849000	1.883141000
	1	0.734771000	1.250202000	4.850742000
	1	3.698836000	-0.143685000	4.809219000
	1	5.858855000	0.974730000	4.575172000
	1	6.035617000	3.190695000	3.451351000
	1	0.288167000	-1.158287000	4.509001000
	1	1.726854000	-1.316850000	3.497030000
	1	1.866779000	-0.933208000	5.223806000

Table S3. The Computational Workfllow

5.1. File espera_A1.mol - produced by program **ChemSketch** by 3D-optimization from a figure in **ChemDraw**:

```
  ACD/Labs08092508183D

170175  0  0  0  0  0  0  0  0  1 V2000
   20.5866   -7.9900   -3.3852 C   0  0  0  0  0  0  0  0  0  0  0  0
   19.9130   -6.7013   -3.1978 C   0  0  0  0  0  0  0  0  0  0  0  0
   19.8012   -9.1309   -3.3547 C   0  0  0  0  0  0  0  0  0  0  0  0
   22.0899   -7.7327   -3.7128 C   0  0  0  0  0  0  0  0  0  0  0  0
   19.1494   -6.9842   -4.4440 C   0  0  0  0  0  0  0  0  0  0  0  0
   19.3778   -6.4463   -1.9825 C   0  0  0  0  0  0  0  0  0  0  0  0
   18.2415   -8.8755   -3.3266 C   0  0  0  0  0  0  0  0  0  0  0  0
   20.2852  -10.4172   -3.4879 N   0  0  0  0  0  0  0  0  0  0  0  0
   22.6668   -8.8529   -4.3945 O   0  0  0  0  0  0  0  0  0  0  0  0
   22.1389   -6.4781   -4.5886 C   0  0  0  0  0  0  0  0  0  0  0  0
   17.8426   -7.5734   -4.0661 C   0  0  0  0  0  0  0  0  0  0  0  0
   19.1402   -5.7879   -5.1396 C   0  0  0  0  0  0  0  0  0  0  0  0
   19.7771   -7.9543   -5.2701 O   0  0  0  0  0  0  0  0  0  0  0  0
   19.1839   -7.5388   -0.9774 C   0  0  0  0  0  0  0  0  0  0  0  0
   17.4461   -9.6189   -2.7985 O   0  0  0  0  0  0  0  0  0  0  0  0
   19.3644  -11.4512   -3.4537 C   0  0  0  0  0  0  0  0  0  0  0  0
   23.9720   -9.1450   -3.8678 C   0  0  0  0  0  0  0  0  0  0  0  0
   22.0208   -5.4384   -5.2531 C   0  0  0  0  0  0  0  0  0  0  0  0
   17.0743   -6.7106   -3.2244 O   0  0  0  0  0  0  0  0  0  0  0  0
   19.5502   -4.8199   -5.6953 C   0  0  0  0  0  0  0  0  0  0  0  0
   20.7051   -7.6400    0.0000 S   0  0  0  0  0  0  0  0  0  0  0  0
   19.7212  -12.7155   -3.7929 O   0  0  0  0  0  0  0  0  0  0  0  0
   18.2181  -11.2172   -3.1400 O   0  0  0  0  0  0  0  0  0  0  0  0
   24.3190  -10.6257   -4.0922 C   0  0  0  0  0  0  0  0  0  0  0  0
   24.9421   -8.3330   -4.5296 O   0  0  0  0  0  0  0  0  0  0  0  0
   21.7106   -4.1603   -6.0244 C   0  0  0  0  0  0  0  0  0  0  0  0
   15.6589   -6.9734   -3.0977 C   0  0  0  0  0  0  0  0  0  0  0  0
   20.3925   -3.8399   -6.2503 C   0  0  0  0  0  0  0  0  0  0  0  0
   22.1546   -7.7347   -1.4120 S   0  0  0  0  0  0  0  0  0  0  0  0
   19.9283  -12.9798   -5.1702 C   0  0  0  0  0  0  0  0  0  0  0  0
   23.3696  -11.4556   -3.4139 O   0  0  0  0  0  0  0  0  0  0  0  0
   25.7302  -10.8895   -3.5367 C   0  0  0  0  0  0  0  0  0  0  0  0
   26.2641   -8.4828   -4.0010 C   0  0  0  0  0  0  0  0  0  0  0  0
   15.3798   -8.5001   -3.0907 C   0  0  0  0  0  0  0  0  0  0  0  0
   14.9646   -6.3892   -4.1993 O   0  0  0  0  0  0  0  0  0  0  0  0
   22.6025   -5.8329   -1.9422 S   0  0  0  0  0  0  0  0  0  0  0  0
   23.2240  -12.8181   -3.8390 C   0  0  0  0  0  0  0  0  0  0  0  0
   26.7326   -9.9372   -4.1808 C   0  0  0  0  0  0  0  0  0  0  0  0
   26.1171  -12.2235   -3.8256 O   0  0  0  0  0  0  0  0  0  0  0  0
   27.2208   -7.5464   -4.7460 C   0  0  0  0  0  0  0  0  0  0  0  0
   13.9991   -8.8011   -3.7074 C   0  0  0  0  0  0  0  0  0  0  0  0
   13.5309   -6.3700   -4.0683 C   0  0  0  0  0  0  0  0  0  0  0  0
   24.2947   -5.4970   -1.3884 C   0  0  0  0  0  0  0  0  0  0  0  0
   24.5941  -13.5063   -3.8854 C   0  0  0  0  0  0  0  0  0  0  0  0
```

22.3822	-13.5047	-2.9135	O	0	0	0	0	0	0	0	0	0	0	0	0
28.0462	-10.1215	-3.5350	N	0	0	0	0	0	0	0	0	0	0	0	0
13.4848	-10.0237	-3.1767	O	0	0	0	0	0	0	0	0	0	0	0	0
13.0493	-7.6568	-3.3754	C	0	0	0	0	0	0	0	0	0	0	0	0
13.1032	-5.1635	-3.2295	C	0	0	0	0	0	0	0	0	0	0	0	0
24.5814	-14.9217	-3.3045	C	0	0	0	0	0	0	0	0	0	0	0	0
22.0917	-14.8750	-3.1998	C	0	0	0	0	0	0	0	0	0	0	0	0
29.0628	-10.4874	-4.5199	O	0	0	0	0	0	0	0	0	0	0	0	0
12.4291	-10.6829	-3.7504	C	0	0	0	0	0	0	0	0	0	0	0	0
13.0272	-7.4547	-1.9689	O	0	0	0	0	0	0	0	0	0	0	0	0
23.3064	-15.6380	-3.7319	C	0	0	0	0	0	0	0	0	0	0	0	0
25.7141	-15.6436	-3.7856	O	0	0	0	0	0	0	0	0	0	0	0	0
30.1698	-11.1130	-3.8828	C	0	0	0	0	0	0	0	0	0	0	0	0
12.3123	-12.1518	-3.5973	C	0	0	0	0	0	0	0	0	0	0	0	0
11.6156	-10.0750	-4.4097	O	0	0	0	0	0	0	0	0	0	0	0	0
23.2979	-17.0086	-3.1868	N	0	0	0	0	0	0	0	0	0	0	0	0
26.9432	-15.1061	-3.3060	C	0	0	0	0	0	0	0	0	0	0	0	0
30.4346	-12.4788	-4.5287	C	0	0	0	0	0	0	0	0	0	0	0	0
31.3252	-10.2908	-4.0274	O	0	0	0	0	0	0	0	0	0	0	0	0
11.0789	-12.7959	-3.5254	C	0	0	0	0	0	0	0	0	0	0	0	0
13.4814	-12.8932	-3.5298	C	0	0	0	0	0	0	0	0	0	0	0	0
24.1097	-17.9434	-3.9839	C	0	0	0	0	0	0	0	0	0	0	0	0
31.6794	-13.1298	-3.9276	C	0	0	0	0	0	0	0	0	0	0	0	0
32.4691	-10.8242	-3.3585	C	0	0	0	0	0	0	0	0	0	0	0	0
11.0404	-14.1780	-3.3889	C	0	0	0	0	0	0	0	0	0	0	0	0
9.8587	-12.0743	-3.5923	N	0	0	0	0	0	0	0	0	0	0	0	0
13.4351	-14.2714	-3.3923	C	0	0	0	0	0	0	0	0	0	0	0	0
25.5791	-17.8971	-3.5615	C	0	0	0	0	0	0	0	0	0	0	0	0
23.5645	-19.3591	-3.7964	C	0	0	0	0	0	0	0	0	0	0	0	0
32.8626	-12.1588	-4.0096	C	0	0	0	0	0	0	0	0	0	0	0	0
31.4289	-13.4529	-2.5662	O	0	0	0	0	0	0	0	0	0	0	0	0
33.6292	-9.8409	-3.4669	C	0	0	0	0	0	0	0	0	0	0	0	0
12.2107	-14.9150	-3.3234	C	0	0	0	0	0	0	0	0	0	0	0	0
8.6571	-12.7688	-3.5231	C	0	0	0	0	0	0	0	0	0	0	0	0
14.5831	-15.0031	-3.3211	O	0	0	0	0	0	0	0	0	0	0	0	0
34.2850	-12.8700	-3.1421	S	0	0	0	0	0	0	0	0	0	0	0	0
12.1483	-16.2725	-3.1883	O	0	0	0	0	0	0	0	0	0	0	0	0
7.3366	-12.1067	-3.5954	C	0	0	0	0	0	0	0	0	0	0	0	0
8.6914	-13.9722	-3.3974	O	0	0	0	0	0	0	0	0	0	0	0	0
15.6874	-14.7566	-4.1804	C	0	0	0	0	0	0	0	0	0	0	0	0
35.7872	-12.2503	-3.9435	C	0	0	0	0	0	0	0	0	0	0	0	0
12.8082	-17.1374	-4.1012	C	0	0	0	0	0	0	0	0	0	0	0	0
6.2183	-12.8922	-3.5071	O	0	0	0	0	0	0	0	0	0	0	0	0
7.2332	-10.7759	-3.7418	C	0	0	0	0	0	0	0	0	0	0	0	0
4.8775	-12.4200	-3.5808	C	0	0	0	0	0	0	0	0	0	0	0	0
22.6664	-7.5765	-2.7738	H	0	0	0	0	0	0	0	0	0	0	0	0
20.1189	-5.9598	-1.3086	H	0	0	0	0	0	0	0	0	0	0	0	0
17.2111	-7.7736	-4.9635	H	0	0	0	0	0	0	0	0	0	0	0	0
23.9806	-8.9315	-2.7766	H	0	0	0	0	0	0	0	0	0	0	0	0
24.2892	-10.8581	-5.1825	H	0	0	0	0	0	0	0	0	0	0	0	0
15.3001	-6.5311	-2.1413	H	0	0	0	0	0	0	0	0	0	0	0	0
25.7201	-10.7283	-2.4345	H	0	0	0	0	0	0	0	0	0	0	0	0
26.2573	-8.2229	-2.9178	H	0	0	0	0	0	0	0	0	0	0	0	0
22.7698	-12.8408	-4.8558	H	0	0	0	0	0	0	0	0	0	0	0	0
26.8126	-10.1605	-5.2703	H	0	0	0	0	0	0	0	0	0	0	0	0
14.0992	-8.9002	-4.8128	H	0	0	0	0	0	0	0	0	0	0	0	0
13.0756	-6.2991	-5.0832	H	0	0	0	0	0	0	0	0	0	0	0	0

12.0238	-7.9090	-3.7350	H	0	0	0	0	0	0	0	0	0	0	0	0
24.6186	-14.8657	-2.1929	H	0	0	0	0	0	0	0	0	0	0	0	0
23.2624	-15.6778	-4.8454	H	0	0	0	0	0	0	0	0	0	0	0	0
29.9413	-11.2539	-2.8011	H	0	0	0	0	0	0	0	0	0	0	0	0
31.9227	-14.0568	-4.4973	H	0	0	0	0	0	0	0	0	0	0	0	0
32.2250	-10.9886	-2.2834	H	0	0	0	0	0	0	0	0	0	0	0	0
33.1255	-11.9837	-5.0788	H	0	0	0	0	0	0	0	0	0	0	0	0
21.2623	-10.5950	-3.6636	H	0	0	0	0	0	0	0	0	0	0	0	0
19.8708	-7.5195	-6.1202	H	0	0	0	0	0	0	0	0	0	0	0	0
18.3377	-7.2719	-0.3034	H	0	0	0	0	0	0	0	0	0	0	0	0
18.9664	-8.5044	-1.4881	H	0	0	0	0	0	0	0	0	0	0	0	0
22.5278	-3.5014	-6.3858	H	0	0	0	0	0	0	0	0	0	0	0	0
20.0436	-2.9342	-6.7899	H	0	0	0	0	0	0	0	0	0	0	0	0
19.0523	-12.6314	-5.7571	H	0	0	0	0	0	0	0	0	0	0	0	0
20.8348	-12.4330	-5.5045	H	0	0	0	0	0	0	0	0	0	0	0	0
20.0592	-14.0688	-5.3424	H	0	0	0	0	0	0	0	0	0	0	0	0
16.1548	-9.0190	-3.6979	H	0	0	0	0	0	0	0	0	0	0	0	0
15.4203	-8.8772	-2.0448	H	0	0	0	0	0	0	0	0	0	0	0	0
27.0661	-12.2322	-3.6338	H	0	0	0	0	0	0	0	0	0	0	0	0
27.1498	-7.7225	-5.8415	H	0	0	0	0	0	0	0	0	0	0	0	0
26.9383	-6.4949	-4.5223	H	0	0	0	0	0	0	0	0	0	0	0	0
28.2732	-7.7160	-4.4325	H	0	0	0	0	0	0	0	0	0	0	0	0
24.9923	-5.4697	-2.2551	H	0	0	0	0	0	0	0	0	0	0	0	0
24.3107	-4.5087	-0.8789	H	0	0	0	0	0	0	0	0	0	0	0	0
24.6460	-6.2810	-0.6841	H	0	0	0	0	0	0	0	0	0	0	0	0
24.9295	-13.5507	-4.9471	H	0	0	0	0	0	0	0	0	0	0	0	0
25.3004	-12.8962	-3.2920	H	0	0	0	0	0	0	0	0	0	0	0	0
28.3198	-9.2236	-3.1722	H	0	0	0	0	0	0	0	0	0	0	0	0
13.0354	-4.2548	-3.8660	H	0	0	0	0	0	0	0	0	0	0	0	0
12.1079	-5.3763	-2.7826	H	0	0	0	0	0	0	0	0	0	0	0	0
13.8379	-4.9740	-2.4180	H	0	0	0	0	0	0	0	0	0	0	0	0
21.7638	-15.3610	-2.2543	H	0	0	0	0	0	0	0	0	0	0	0	0
21.2715	-14.9348	-3.9459	H	0	0	0	0	0	0	0	0	0	0	0	0
12.1957	-7.8340	-1.6712	H	0	0	0	0	0	0	0	0	0	0	0	0
23.7419	-16.9541	-2.2822	H	0	0	0	0	0	0	0	0	0	0	0	0
27.5135	-14.6532	-4.1457	H	0	0	0	0	0	0	0	0	0	0	0	0
26.7295	-14.3294	-2.5417	H	0	0	0	0	0	0	0	0	0	0	0	0
27.5648	-15.9068	-2.8485	H	0	0	0	0	0	0	0	0	0	0	0	0
29.5578	-13.1403	-4.3435	H	0	0	0	0	0	0	0	0	0	0	0	0
30.5745	-12.3481	-5.6243	H	0	0	0	0	0	0	0	0	0	0	0	0
14.4615	-12.3766	-3.5869	H	0	0	0	0	0	0	0	0	0	0	0	0
24.0329	-17.6406	-5.0537	H	0	0	0	0	0	0	0	0	0	0	0	0
10.0613	-14.6985	-3.3330	H	0	0	0	0	0	0	0	0	0	0	0	0
9.8752	-11.0729	-3.6966	H	0	0	0	0	0	0	0	0	0	0	0	0
26.2376	-18.1327	-4.4252	H	0	0	0	0	0	0	0	0	0	0	0	0
25.8051	-16.8745	-3.1915	H	0	0	0	0	0	0	0	0	0	0	0	0
25.7817	-18.6315	-2.7529	H	0	0	0	0	0	0	0	0	0	0	0	0
23.5305	-19.8936	-4.7694	H	0	0	0	0	0	0	0	0	0	0	0	0
24.2225	-19.9161	-3.0940	H	0	0	0	0	0	0	0	0	0	0	0	0
22.5362	-19.3187	-3.3779	H	0	0	0	0	0	0	0	0	0	0	0	0
31.7471	-14.3548	-2.4569	H	0	0	0	0	0	0	0	0	0	0	0	0
33.9873	-9.7799	-4.5168	H	0	0	0	0	0	0	0	0	0	0	0	0
33.2785	-8.8385	-3.1368	H	0	0	0	0	0	0	0	0	0	0	0	0
34.4674	-10.1647	-2.8157	H	0	0	0	0	0	0	0	0	0	0	0	0
15.3675	-14.8214	-5.2429	H	0	0	0	0	0	0	0	0	0	0	0	0
16.0855	-13.7396	-3.9755	H	0	0	0	0	0	0	0	0	0	0	0	0
16.4858	-15.5077	-3.9996	H	0	0	0	0	0	0	0	0	0	0	0	0

```
   35.6980  -12.3078   -5.0499 H   0  0  0  0  0  0  0  0  0  0  0  0
   35.9501  -11.1926   -3.6449 H   0  0  0  0  0  0  0  0  0  0  0  0
   36.6694  -12.8509   -3.6326 H   0  0  0  0  0  0  0  0  0  0  0  0
   12.5375  -16.8713   -5.1456 H   0  0  0  0  0  0  0  0  0  0  0  0
   13.9063  -17.0320   -3.9654 H   0  0  0  0  0  0  0  0  0  0  0  0
   12.5117  -18.1910   -3.9102 H   0  0  0  0  0  0  0  0  0  0  0  0
    6.2298  -10.3019   -3.7957 H   0  0  0  0  0  0  0  0  0  0  0  0
    8.1424  -10.1445   -3.8096 H   0  0  0  0  0  0  0  0  0  0  0  0
    4.6332  -12.1180   -4.6218 H   0  0  0  0  0  0  0  0  0  0  0  0
    4.1921  -13.2358   -3.2636 H   0  0  0  0  0  0  0  0  0  0  0  0
    4.7398  -11.5447   -2.9099 H   0  0  0  0  0  0  0  0  0  0  0  0
  1  2  1  0  0  0  0
  1  3  2  0  0  0  0
  1  4  1  0  0  0  0
  2  5  1  0  0  0  0
  2  6  2  0  0  0  0
  3  7  1  0  0  0  0
  3  8  1  0  0  0  0
  4  9  1  0  0  0  0
  4 10  1  0  0  0  0
  5 11  1  0  0  0  0
  5 12  1  0  0  0  0
  5 13  1  0  0  0  0
  6 14  1  0  0  0  0
  7 15  2  0  0  0  0
  7 11  1  0  0  0  0
  8 16  1  0  0  0  0
 17  9  1  0  0  0  0
 10 18  3  0  0  0  0
 11 19  1  0  0  0  0
 12 20  3  0  0  0  0
 14 21  1  0  0  0  0
 16 22  1  0  0  0  0
 16 23  2  0  0  0  0
 17 24  1  0  0  0  0
 17 25  1  0  0  0  0
 18 26  1  0  0  0  0
 27 19  1  0  0  0  0
 20 28  1  0  0  0  0
 21 29  1  0  0  0  0
 22 30  1  0  0  0  0
 24 31  1  0  0  0  0
 24 32  1  0  0  0  0
 25 33  1  0  0  0  0
 26 28  2  0  0  0  0
 27 34  1  0  0  0  0
 27 35  1  0  0  0  0
 29 36  1  0  0  0  0
 37 31  1  0  0  0  0
 32 38  1  0  0  0  0
 32 39  1  0  0  0  0
 33 40  1  0  0  0  0
 33 38  1  0  0  0  0
 34 41  1  0  0  0  0
 35 42  1  0  0  0  0
 36 43  1  0  0  0  0
 37 44  1  0  0  0  0
```

37	45	1	0	0	0	0
38	46	1	0	0	0	0
41	47	1	0	0	0	0
41	48	1	0	0	0	0
42	49	1	0	0	0	0
42	48	1	0	0	0	0
44	50	1	0	0	0	0
45	51	1	0	0	0	0
46	52	1	0	0	0	0
47	53	1	0	0	0	0
48	54	1	0	0	0	0
50	55	1	0	0	0	0
50	56	1	0	0	0	0
51	55	1	0	0	0	0
57	52	1	0	0	0	0
53	58	1	0	0	0	0
53	59	2	0	0	0	0
55	60	1	0	0	0	0
56	61	1	0	0	0	0
57	62	1	0	0	0	0
57	63	1	0	0	0	0
58	64	2	0	0	0	0
58	65	1	0	0	0	0
60	66	1	0	0	0	0
62	67	1	0	0	0	0
63	68	1	0	0	0	0
64	69	1	0	0	0	0
64	70	1	0	0	0	0
65	71	2	0	0	0	0
66	72	1	0	0	0	0
66	73	1	0	0	0	0
67	74	1	0	0	0	0
67	75	1	0	0	0	0
68	76	1	0	0	0	0
68	74	1	0	0	0	0
69	77	2	0	0	0	0
70	78	1	0	0	0	0
71	79	1	0	0	0	0
71	77	1	0	0	0	0
74	80	1	0	0	0	0
77	81	1	0	0	0	0
78	82	1	0	0	0	0
78	83	2	0	0	0	0
79	84	1	0	0	0	0
80	85	1	0	0	0	0
81	86	1	0	0	0	0
82	87	1	0	0	0	0
82	88	2	0	0	0	0
87	89	1	0	0	0	0
90	4	1	0	0	0	0
91	6	1	0	0	0	0
92	11	1	0	0	0	0
93	17	1	0	0	0	0
94	24	1	0	0	0	0
95	27	1	0	0	0	0
96	32	1	0	0	0	0
97	33	1	0	0	0	0

98	37	1	0	0	0	0
99	38	1	0	0	0	0
100	41	1	0	0	0	0
101	42	1	0	0	0	0
102	48	1	0	0	0	0
103	50	1	0	0	0	0
104	55	1	0	0	0	0
105	57	1	0	0	0	0
106	67	1	0	0	0	0
107	68	1	0	0	0	0
108	74	1	0	0	0	0
109	8	1	0	0	0	0
110	13	1	0	0	0	0
111	14	1	0	0	0	0
112	14	1	0	0	0	0
113	26	1	0	0	0	0
114	28	1	0	0	0	0
115	30	1	0	0	0	0
116	30	1	0	0	0	0
117	30	1	0	0	0	0
118	34	1	0	0	0	0
119	34	1	0	0	0	0
120	39	1	0	0	0	0
121	40	1	0	0	0	0
122	40	1	0	0	0	0
123	40	1	0	0	0	0
124	43	1	0	0	0	0
125	43	1	0	0	0	0
126	43	1	0	0	0	0
127	44	1	0	0	0	0
128	44	1	0	0	0	0
129	46	1	0	0	0	0
130	49	1	0	0	0	0
131	49	1	0	0	0	0
132	49	1	0	0	0	0
133	51	1	0	0	0	0
134	51	1	0	0	0	0
135	54	1	0	0	0	0
136	60	1	0	0	0	0
137	61	1	0	0	0	0
138	61	1	0	0	0	0
139	61	1	0	0	0	0
140	62	1	0	0	0	0
141	62	1	0	0	0	0
142	65	1	0	0	0	0
143	66	1	0	0	0	0
144	69	1	0	0	0	0
145	70	1	0	0	0	0
146	72	1	0	0	0	0
147	72	1	0	0	0	0
148	72	1	0	0	0	0
149	73	1	0	0	0	0
150	73	1	0	0	0	0
151	73	1	0	0	0	0
152	75	1	0	0	0	0
153	76	1	0	0	0	0
154	76	1	0	0	0	0

```
155 76  1  0  0  0  0
156 84  1  0  0  0  0
157 84  1  0  0  0  0
158 84  1  0  0  0  0
159 85  1  0  0  0  0
160 85  1  0  0  0  0
161 85  1  0  0  0  0
162 86  1  0  0  0  0
163 86  1  0  0  0  0
164 86  1  0  0  0  0
165 88  1  0  0  0  0
166 88  1  0  0  0  0
167 89  1  0  0  0  0
168 89  1  0  0  0  0
169 89  1  0  0  0  0
M  END
```

5.2. Edit file espera_A1.mop to obtain input file for **MOPAC** - remove the bonding part of the .mol file

```
  PM7 PRECISE MOZYME XYZ
  ChemDraw08051019392D

 C        20.5866   -7.9900   -3.3852
 C        19.9130   -6.7013   -3.1978
 C        19.8012   -9.1309   -3.3547
 C        22.0899   -7.7327   -3.7128
 C        19.1494   -6.9842   -4.4440
 C        19.3778   -6.4463   -1.9825
 C        18.2415   -8.8755   -3.3266
 N        20.2852  -10.4172   -3.4879
 O        22.6668   -8.8529   -4.3945
 C        22.1389   -6.4781   -4.5886
 C        17.8426   -7.5734   -4.0661
 C        19.1402   -5.7879   -5.1396
 O        19.7771   -7.9543   -5.2701
 C        19.1839   -7.5388   -0.9774
 O        17.4461   -9.6189   -2.7985
 C        19.3644  -11.4512   -3.4537
 C        23.9720   -9.1450   -3.8678
 C        22.0208   -5.4384   -5.2531
 O        17.0743   -6.7106   -3.2244
 C        19.5502   -4.8199   -5.6953
 S        20.7051   -7.6400    0.0000
 O        19.7212  -12.7155   -3.7929
 O        18.2181  -11.2172   -3.1400
 C        24.3190  -10.6257   -4.0922
 O        24.9421   -8.3330   -4.5296
 C        21.7106   -4.1603   -6.0244
 C        15.6589   -6.9734   -3.0977
 C        20.3925   -3.8399   -6.2503
 S        22.1546   -7.7347   -1.4120
 C        19.9283  -12.9798   -5.1702
 O        23.3696  -11.4556   -3.4139
 C        25.7302  -10.8895   -3.5367
```

C	26.2641	-8.4828	-4.0010
C	15.3798	-8.5001	-3.0907
O	14.9646	-6.3892	-4.1993
S	22.6025	-5.8329	-1.9422
C	23.2240	-12.8181	-3.8390
C	26.7326	-9.9372	-4.1808
O	26.1171	-12.2235	-3.8256
C	27.2208	-7.5464	-4.7460
C	13.9991	-8.8011	-3.7074
C	13.5309	-6.3700	-4.0683
C	24.2947	-5.4970	-1.3884
C	24.5941	-13.5063	-3.8854
O	22.3822	-13.5047	-2.9135
N	28.0462	-10.1215	-3.5350
O	13.4848	-10.0237	-3.1767
C	13.0493	-7.6568	-3.3754
C	13.1032	-5.1635	-3.2295
C	24.5814	-14.9217	-3.3045
C	22.0917	-14.8750	-3.1998
O	29.0628	-10.4874	-4.5199
C	12.4291	-10.6829	-3.7504
O	13.0272	-7.4547	-1.9689
C	23.3064	-15.6380	-3.7319
O	25.7141	-15.6436	-3.7856
C	30.1698	-11.1130	-3.8828
C	12.3123	-12.1518	-3.5973
O	11.6156	-10.0750	-4.4097
N	23.2979	-17.0086	-3.1868
C	26.9432	-15.1061	-3.3060
C	30.4346	-12.4788	-4.5287
O	31.3252	-10.2908	-4.0274
C	11.0789	-12.7959	-3.5254
C	13.4814	-12.8932	-3.5298
C	24.1097	-17.9434	-3.9839
C	31.6794	-13.1298	-3.9276
C	32.4691	-10.8242	-3.3585
C	11.0404	-14.1780	-3.3889
N	9.8587	-12.0743	-3.5923
C	13.4351	-14.2714	-3.3923
C	25.5791	-17.8971	-3.5615
C	23.5645	-19.3591	-3.7964
C	32.8626	-12.1588	-4.0096
O	31.4289	-13.4529	-2.5662
C	33.6292	-9.8409	-3.4669
C	12.2107	-14.9150	-3.3234
C	8.6571	-12.7688	-3.5231
O	14.5831	-15.0031	-3.3211
S	34.2850	-12.8700	-3.1421
O	12.1483	-16.2725	-3.1883
C	7.3366	-12.1067	-3.5954
O	8.6914	-13.9722	-3.3974
C	15.6874	-14.7566	-4.1804
C	35.7872	-12.2503	-3.9435
C	12.8082	-17.1374	-4.1012
O	6.2183	-12.8922	-3.5071
C	7.2332	-10.7759	-3.7418
C	4.8775	-12.4200	-3.5808

H	22.6664	-7.5765	-2.7738
H	20.1189	-5.9598	-1.3086
H	17.2111	-7.7736	-4.9635
H	23.9806	-8.9315	-2.7766
H	24.2892	-10.8581	-5.1825
H	15.3001	-6.5311	-2.1413
H	25.7201	-10.7283	-2.4345
H	26.2573	-8.2229	-2.9178
H	22.7698	-12.8408	-4.8558
H	26.8126	-10.1605	-5.2703
H	14.0992	-8.9002	-4.8128
H	13.0756	-6.2991	-5.0832
H	12.0238	-7.9090	-3.7350
H	24.6186	-14.8657	-2.1929
H	23.2624	-15.6778	-4.8454
H	29.9413	-11.2539	-2.8011
H	31.9227	-14.0568	-4.4973
H	32.2250	-10.9886	-2.2834
H	33.1255	-11.9837	-5.0788
H	21.2623	-10.5950	-3.6636
H	19.8708	-7.5195	-6.1202
H	18.3377	-7.2719	-0.3034
H	18.9664	-8.5044	-1.4881
H	22.5278	-3.5014	-6.3858
H	20.0436	-2.9342	-6.7899
H	19.0523	-12.6314	-5.7571
H	20.8348	-12.4330	-5.5045
H	20.0592	-14.0688	-5.3424
H	16.1548	-9.0190	-3.6979
H	15.4203	-8.8772	-2.0448
H	27.0661	-12.2322	-3.6338
H	27.1498	-7.7225	-5.8415
H	26.9383	-6.4949	-4.5223
H	28.2732	-7.7160	-4.4325
H	24.9923	-5.4697	-2.2551
H	24.3107	-4.5087	-0.8789
H	24.6460	-6.2810	-0.6841
H	24.9295	-13.5507	-4.9471
H	25.3004	-12.8962	-3.2920
H	28.3198	-9.2236	-3.1722
H	13.0354	-4.2548	-3.8660
H	12.1079	-5.3763	-2.7826
H	13.8379	-4.9740	-2.4180
H	21.7638	-15.3610	-2.2543
H	21.2715	-14.9348	-3.9459
H	12.1957	-7.8340	-1.6712
H	23.7419	-16.9541	-2.2822
H	27.5135	-14.6532	-4.1457
H	26.7295	-14.3294	-2.5417
H	27.5648	-15.9068	-2.8485
H	29.5578	-13.1403	-4.3435
H	30.5745	-12.3481	-5.6243
H	14.4615	-12.3766	-3.5869
H	24.0329	-17.6406	-5.0537
H	10.0613	-14.6985	-3.3330
H	9.8752	-11.0729	-3.6966
H	26.2376	-18.1327	-4.4252

```
 H        25.8051  -16.8745   -3.1915
 H        25.7817  -18.6315   -2.7529
 H        23.5305  -19.8936   -4.7694
 H        24.2225  -19.9161   -3.0940
 H        22.5362  -19.3187   -3.3779
 H        31.7471  -14.3548   -2.4569
 H        33.9873   -9.7799   -4.5168
 H        33.2785   -8.8385   -3.1368
 H        34.4674  -10.1647   -2.8157
 H        15.3675  -14.8214   -5.2429
 H        16.0855  -13.7396   -3.9755
 H        16.4858  -15.5077   -3.9996
 H        35.6980  -12.3078   -5.0499
 H        35.9501  -11.1926   -3.6449
 H        36.6694  -12.8509   -3.6326
 H        12.5375  -16.8713   -5.1456
 H        13.9063  -17.0320   -3.9654
 H        12.5117  -18.1910   -3.9102
 H         6.2298  -10.3019   -3.7957
 H         8.1424  -10.1445   -3.8096
 H         4.6332  -12.1180   -4.6218
 H         4.1921  -13.2358   -3.2636
 H         4.7398  -11.5447   -2.9099
```

5.3 **MOPAC** produces the file **espera_A1.arc**

with semiempirical QM energies

```
                     SUMMARY OF  PM7 CALCULATION

                                                       MOPAC v22.0.6 Linux
                                                       Tue Mar 31 20:27:53
2026

           Empirical Formula: C59 H80 N4 O22 S4  =   169 atoms

 PM7 PRECISE MOZYME XYZ
  ChemDraw08051019392D



     HERBERTS TEST WAS SATISFIED IN BFGS
     SCF FIELD WAS ACHIEVED

          HEAT OF FORMATION       =       -690.03154 KCAL/MOL =   -2887.09198
KJ/MOL
          GRADIENT NORM           =          9.55810          =       0.73524
PER ATOM
          DIPOLE                  =         14.49085 DEBYE   POINT GROUP:  C1
          NO. OF FILLED LEVELS    =        246
          MOLECULAR WEIGHT        =       1325.5346

          MOLECULAR DIMENSIONS (Angstroms)
```

```
  Atom       Atom       Distance
   H   169    H   161    30.70403
   H   114    H   164    16.64079
   S    29    H   116     9.09656
 SCF CALCULATIONS        =        671
 WALL-CLOCK TIME         =          5 MINUTES AND 14.148 SECONDS
 COMPUTATION TIME        =         19 MINUTES AND 58.133 SECONDS


 FINAL GEOMETRY OBTAINED
PM7 PRECISE MOZYME XYZ
 ChemDraw08051019392D

 C    20.76707048 +1  -8.34525320 +1  -3.00118239 +1
 C    19.77560203 +1  -7.29192006 +1  -2.82253994 +1
 C    20.24489522 +1  -9.48198832 +1  -3.52629324 +1
 C    22.19193354 +1  -7.86938616 +1  -2.88755112 +1
 C    19.09060264 +1  -7.10268667 +1  -4.19364308 +1
 C    19.39741487 +1  -6.64270312 +1  -1.71957430 +1
 C    18.72531339 +1  -9.51834107 +1  -3.71139554 +1
 N    20.96969612 +1 -10.60809621 +1  -3.90328432 +1
 O    22.89643508 +1  -8.64936950 +1  -3.85385801 +1
 C    22.23626095 +1  -6.46295643 +1  -3.24879986 +1
 C    18.00935085 +1  -8.20875474 +1  -4.18671719 +1
 C    19.04450086 +1  -5.67750705 +1  -4.37880694 +1
 O    19.88312544 +1  -7.56837355 +1  -5.28820998 +1
 C    20.00226771 +1  -6.85351687 +1  -0.38397185 +1
 O    18.09131090 +1 -10.48217950 +1  -3.39747819 +1
 C    20.52551213 +1 -11.37574254 +1  -4.97342368 +1
 C    24.27717294 +1  -8.75156405 +1  -3.64963838 +1
 C    22.11548197 +1  -5.29565864 +1  -3.57090006 +1
 O    17.11487045 +1  -7.87944501 +1  -3.15418012 +1
 C    19.61835226 +1  -4.60295501 +1  -4.42405831 +1
 S    20.63768727 +1  -5.22865092 +1   0.19696052 +1
 O    21.49400605 +1 -12.28933143 +1  -5.29646806 +1
 O    19.47167646 +1 -11.26723891 +1  -5.55515811 +1
 C    24.62112513 +1 -10.13329542 +1  -4.25060122 +1
 O    24.85166280 +1  -7.70214913 +1  -4.40782831 +1
 C    21.83271027 +1  -3.96254770 +1  -3.93239351 +1
 C    15.75254953 +1  -8.12222831 +1  -3.35429856 +1
 C    20.59198172 +1  -3.59282305 +1  -4.34199210 +1
 S    22.50216930 +1  -5.34830779 +1   0.83137112 +1
 C    21.13984129 +1 -13.15881533 +1  -6.36996302 +1
 O    24.29108447 +1 -10.98565783 +1  -3.16927541 +1
 C    26.12306536 +1 -10.26829566 +1  -4.52822499 +1
 C    26.26847954 +1  -7.72434656 +1  -4.41258775 +1
 C    15.41446469 +1  -9.55451195 +1  -3.77509195 +1
 O    15.31342843 +1  -7.25888048 +1  -4.39491768 +1
 S    24.01601901 +1  -4.73699070 +1  -0.28577003 +1
 C    24.10181216 +1 -12.35035216 +1  -3.51106287 +1
 C    26.75065839 +1  -9.00198220 +1  -5.15346455 +1
 O    26.20721017 +1 -11.33370094 +1  -5.45318152 +1
 C    26.64010077 +1  -6.44445236 +1  -5.15326150 +1
 C    14.00118019 +1  -9.59322531 +1  -4.36258111 +1
 C    13.90807715 +1  -7.07672300 +1  -4.40353253 +1
 C    24.83058192 +1  -6.13536676 +1  -1.10623550 +1
```

C	25.34249840	+1	-13.13161319	+1	-3.07022896	+1
O	23.00406434	+1	-12.68808678	+1	-2.67411729	+1
N	28.21776298	+1	-9.07678716	+1	-4.93127409	+1
O	13.47127692	+1	-10.78116862	+1	-3.76630032	+1
C	13.16614051	+1	-8.36234009	+1	-3.96105911	+1
C	13.54022698	+1	-5.87721517	+1	-3.54153595	+1
C	24.95307917	+1	-14.51191322	+1	-2.53176095	+1
C	22.51877785	+1	-13.99495022	+1	-2.90506327	+1
O	28.67889502	+1	-10.24069902	+1	-5.60561317	+1
C	12.30286527	+1	-11.29748424	+1	-4.22066625	+1
O	13.04515422	+1	-8.29900478	+1	-2.55289779	+1
C	23.64786769	+1	-15.01728552	+1	-3.20274263	+1
O	25.92199252	+1	-15.47714206	+1	-2.91452574	+1
C	29.44283955	+1	-11.00461010	+1	-4.67224151	+1
C	12.16500039	+1	-12.68943372	+1	-3.79924668	+1
O	11.52216625	+1	-10.63136190	+1	-4.86578510	+1
N	23.15550524	+1	-16.30115597	+1	-2.67772324	+1
C	27.17908711	+1	-15.32580468	+1	-2.28385689	+1
C	29.56204826	+1	-12.38833209	+1	-5.32436723	+1
O	30.70858231	+1	-10.36818348	+1	-4.62934283	+1
C	10.90263118	+1	-13.30891845	+1	-3.67242641	+1
C	13.33593545	+1	-13.42254650	+1	-3.54069793	+1
C	23.23694430	+1	-17.43961854	+1	-3.62072120	+1
C	30.59288562	+1	-13.20432411	+1	-4.52850017	+1
C	31.63311065	+1	-11.04594798	+1	-3.78117162	+1
C	10.83725596	+1	-14.65829918	+1	-3.29722606	+1
N	9.75210367	+1	-12.54976118	+1	-3.93909213	+1
C	13.27121732	+1	-14.75071798	+1	-3.17169215	+1
C	24.58309919	+1	-18.17892763	+1	-3.55492227	+1
C	22.08778528	+1	-18.39978677	+1	-3.25930825	+1
C	31.91387929	+1	-12.41859713	+1	-4.42321731	+1
O	29.97799551	+1	-13.35866296	+1	-3.26188739	+1
C	32.83299744	+1	-10.10490773	+1	-3.75787573	+1
C	12.00476690	+1	-15.38034418	+1	-3.05156259	+1
C	8.46563424	+1	-12.87332907	+1	-3.51358109	+1
O	14.35056664	+1	-15.55230233	+1	-2.92245251	+1
S	33.14540664	+1	-13.38396105	+1	-3.46395672	+1
O	11.75321821	+1	-16.66373208	+1	-2.72225903	+1
C	7.44995540	+1	-11.80894485	+1	-3.85272344	+1
O	8.18773499	+1	-13.87482626	+1	-2.90329174	+1
C	15.62297612	+1	-14.91624692	+1	-2.90889558	+1
C	34.68131930	+1	-13.11099123	+1	-4.40119208	+1
C	12.84881843	+1	-17.53043109	+1	-2.45168369	+1
O	6.69896635	+1	-12.27856349	+1	-4.87699306	+1
C	7.35567896	+1	-10.64795516	+1	-3.20685069	+1
C	5.58696475	+1	-11.47920676	+1	-5.26649224	+1
H	22.61093543	+1	-8.06361849	+1	-1.86640439	+1
H	18.57767296	+1	-5.91287756	+1	-1.76498116	+1
H	17.53035779	+1	-8.33834468	+1	-5.17860165	+1
H	24.57084546	+1	-8.65233303	+1	-2.58355004	+1
H	24.01303311	+1	-10.35265745	+1	-5.15730278	+1
H	15.33528190	+1	-7.80775303	+1	-2.36814058	+1
H	26.66606904	+1	-10.57029700	+1	-3.60050003	+1
H	26.64282827	+1	-7.71473712	+1	-3.36299800	+1
H	23.82882602	+1	-12.47475776	+1	-4.57907668	+1
H	26.46088048	+1	-8.95243378	+1	-6.23214640	+1
H	14.00048250	+1	-9.72469970	+1	-5.46622082	+1

H	13.71942751	+1	-6.87546415	+1	-5.48538437	+1
H	12.13702885	+1	-8.40035599	+1	-4.38355931	+1
H	24.85489384	+1	-14.50742193	+1	-1.42589706	+1
H	23.82526081	+1	-15.08309771	+1	-4.30498578	+1
H	28.98797146	+1	-11.00499793	+1	-3.65986215	+1
H	30.74032093	+1	-14.21274120	+1	-4.97738979	+1
H	31.17840153	+1	-11.15513318	+1	-2.76868253	+1
H	32.30486092	+1	-12.25132012	+1	-5.45515583	+1
H	21.92095903	+1	-10.74653840	+1	-3.53867114	+1
H	20.83608788	+1	-7.33684080	+1	-5.18544082	+1
H	19.23498905	+1	-7.19234245	+1	0.34789043	+1
H	20.77754318	+1	-7.64272962	+1	-0.38004972	+1
H	22.65176581	+1	-3.24252153	+1	-3.85273119	+1
H	20.33692120	+1	-2.56828364	+1	-4.60772351	+1
H	20.28054991	+1	-13.77576697	+1	-6.08647689	+1
H	20.91474613	+1	-12.58012212	+1	-7.27294454	+1
H	22.04783393	+1	-13.76065914	+1	-6.49643884	+1
H	16.14253751	+1	-9.95181569	+1	-4.51011729	+1
H	15.50592279	+1	-10.23917684	+1	-2.90422573	+1
H	27.12610149	+1	-11.37903341	+1	-5.79681819	+1
H	26.33202955	+1	-6.48024145	+1	-6.20632331	+1
H	26.11790313	+1	-5.58147647	+1	-4.71216136	+1
H	27.71435839	+1	-6.24308169	+1	-5.10738927	+1
H	25.91078386	+1	-5.93561581	+1	-1.12637143	+1
H	24.68416929	+1	-7.08979944	+1	-0.59121242	+1
H	24.48898482	+1	-6.23661911	+1	-2.14794322	+1
H	26.03988989	+1	-13.23452494	+1	-3.92781750	+1
H	25.88820665	+1	-12.55752786	+1	-2.29705658	+1
H	28.71986706	+1	-8.33382291	+1	-5.41941307	+1
H	14.13585127	+1	-4.99963736	+1	-3.82399200	+1
H	12.47928632	+1	-5.62442748	+1	-3.63371950	+1
H	13.73478395	+1	-6.06978855	+1	-2.47673582	+1
H	21.99439786	+1	-14.22399170	+1	-1.95685793	+1
H	21.78364838	+1	-13.95868100	+1	-3.72683334	+1
H	12.79725752	+1	-9.18508075	+1	-2.20784588	+1
H	23.57801521	+1	-16.53096127	+1	-1.78256334	+1
H	27.67385676	+1	-14.40072145	+1	-2.60543164	+1
H	27.08412371	+1	-15.36218952	+1	-1.19525762	+1
H	27.74066146	+1	-16.19590016	+1	-2.64677394	+1
H	28.58289586	+1	-12.90863878	+1	-5.31355903	+1
H	29.86873202	+1	-12.29645131	+1	-6.38231083	+1
H	14.30316652	+1	-12.92010949	+1	-3.64298737	+1
H	23.07452605	+1	-17.05989454	+1	-4.66235309	+1
H	9.87437937	+1	-15.17165243	+1	-3.19476454	+1
H	9.90098859	+1	-11.66634112	+1	-4.44861740	+1
H	25.41797988	+1	-17.49726786	+1	-3.76625756	+1
H	24.76022968	+1	-18.60921216	+1	-2.56446785	+1
H	24.62708374	+1	-18.99411047	+1	-4.28179937	+1
H	22.05533001	+1	-19.24970500	+1	-3.94559573	+1
H	22.19252336	+1	-18.78899570	+1	-2.24153104	+1
H	21.12235575	+1	-17.88052129	+1	-3.30672411	+1
H	30.47620159	+1	-13.97169829	+1	-2.68758901	+1
H	33.33658757	+1	-10.05783864	+1	-4.72963226	+1
H	32.49833662	+1	-9.08010300	+1	-3.53163946	+1
H	33.56233344	+1	-10.38867761	+1	-2.99236836	+1
H	15.87854723	+1	-14.52575172	+1	-3.89971056	+1
H	15.66728164	+1	-14.12971726	+1	-2.14788219	+1

```
  H    16.29060497 +1 -15.74547009 +1  -2.64177621 +1
  H    34.63425403 +1 -13.54237750 +1  -5.40641107 +1
  H    34.92577195 +1 -12.04655415 +1  -4.48772047 +1
  H    35.50207708 +1 -13.60239181 +1  -3.86097212 +1
  H    13.48821404 +1 -17.63961019 +1  -3.33414848 +1
  H    13.42077011 +1 -17.17985529 +1  -1.58611687 +1
  H    12.33586421 +1 -18.47499747 +1  -2.22366441 +1
  H     6.61378782 +1  -9.89551932 +1  -3.40578311 +1
  H     8.02062014 +1 -10.36012336 +1  -2.40769759 +1
  H     5.91943740 +1 -10.51302344 +1  -5.65767293 +1
  H     5.13746082 +1 -12.08844394 +1  -6.06278376 +1
  H     4.88595313 +1 -11.35298922 +1  -4.43588756 +1
```

5.4. The **MOPAC** archive file has the coordinates to be input to **GAUSSIAN** or **ORCA** --- the job-input file has to be given the extension **.gjf** (Gaussian Job File) under Windows, or **.com** -- under Linux for **GAUSSIAN**, or the extension **.inp** for **ORCA**. The **espera_A1.gjf** has the form

```
%nprocshared=64
%mem=128GB
%chk=oni_espr.chk
# opt=loose freq oniom(wb97xd/6-31g(d,p):pm7) iop(1/8=1,5/7=26144)

esperamicin_A1.mol ----  ChemDraw08051019392D, Chemical Book

 0,1
  C    20.76707048     -8.34525320     -3.00118239
  C    19.77560203     -7.29192006     -2.82253994
  C    20.24489522     -9.48198832     -3.52629324
  C    22.19193354     -7.86938616     -2.88755112
  C    19.09060264     -7.10268667     -4.19364308
  C    19.39741487     -6.64270312     -1.71957430
  C    18.72531339     -9.51834107     -3.71139554
  N    20.96969612    -10.60809621     -3.90328432
  O    22.89643508     -8.64936950     -3.85385801
  C    22.23626095     -6.46295643     -3.24879986
  C    18.00935085     -8.20875474     -4.18671719
  C    19.04450086     -5.67750705     -4.37880694
  O    19.88312544     -7.56837355     -5.28820998
  C    20.00226771     -6.85351687     -0.38397185
  O    18.09131090    -10.48217950     -3.39747819
  C    20.52551213    -11.37574254     -4.97342368
  C    24.27717294     -8.75156405     -3.64963838
  C    22.11548197     -5.29565864     -3.57090006
  O    17.11487045     -7.87944501     -3.15418012
  C    19.61835226     -4.60295501     -4.42405831
  S    20.63768727     -5.22865092      0.19696052
  O    21.49400605    -12.28933143     -5.29646806
  O    19.47167646    -11.26723891     -5.55515811
  C    24.62112513    -10.13329542     -4.25060122
  O    24.85166280     -7.70214913     -4.40782831
  C    21.83271027     -3.96254770     -3.93239351
  C    15.75254953     -8.12222831     -3.35429856
  C    20.59198172     -3.59282305     -4.34199210
```

S	22.50216930	-5.34830779	0.83137112
C	21.13984129	-13.15881533	-6.36996302
O	24.29108447	-10.98565783	-3.16927541
C	26.12306536	-10.26829566	-4.52822499
C	26.26847954	-7.72434656	-4.41258775
C	15.41446469	-9.55451195	-3.77509195
O	15.31342843	-7.25888048	-4.39491768
S	24.01601901	-4.73699070	-0.28577003
C	24.10181216	-12.35035216	-3.51106287
C	26.75065839	-9.00198220	-5.15346455
O	26.20721017	-11.33370094	-5.45318152
C	26.64010077	-6.44445236	-5.15326150
C	14.00118019	-9.59322531	-4.36258111
C	13.90807715	-7.07672300	-4.40353253
C	24.83058192	-6.13536676	-1.10623550
C	25.34249840	-13.13161319	-3.07022896
O	23.00406434	-12.68808678	-2.67411729
N	28.21776298	-9.07678716	-4.93127409
O	13.47127692	-10.78116862	-3.76630032
C	13.16614051	-8.36234009	-3.96105911
C	13.54022698	-5.87721517	-3.54153595
C	24.95307917	-14.51191322	-2.53176095
C	22.51877785	-13.99495022	-2.90506327
O	28.67889502	-10.24069902	-5.60561317
C	12.30286527	-11.29748424	-4.22066625
O	13.04515422	-8.29900478	-2.55289779
C	23.64786769	-15.01728552	-3.20274263
O	25.92199252	-15.47714206	-2.91452574
C	29.44283955	-11.00461010	-4.67224151
C	12.16500039	-12.68943372	-3.79924668
O	11.52216625	-10.63136190	-4.86578510
N	23.15550524	-16.30115597	-2.67772324
C	27.17908711	-15.32580468	-2.28385689
C	29.56204826	-12.38833209	-5.32436723
O	30.70858231	-10.36818348	-4.62934283
C	10.90263118	-13.30891845	-3.67242641
C	13.33593545	-13.42254650	-3.54069793
C	23.23694430	-17.43961854	-3.62072120
C	30.59288562	-13.20432411	-4.52850017
C	31.63311065	-11.04594798	-3.78117162
C	10.83725596	-14.65829918	-3.29722606
N	9.75210367	-12.54976118	-3.93909213
C	13.27121732	-14.75071798	-3.17169215
C	24.58309919	-18.17892763	-3.55492227
C	22.08778528	-18.39978677	-3.25930825
C	31.91387929	-12.41859713	-4.42321731
O	29.97799551	-13.35866296	-3.26188739
C	32.83299744	-10.10490773	-3.75787573
C	12.00476690	-15.38034418	-3.05156259
C	8.46563424	-12.87332907	-3.51358109
O	14.35056664	-15.55230233	-2.92245251
S	33.14540664	-13.38396105	-3.46395672
O	11.75321821	-16.66373208	-2.72225903
C	7.44995540	-11.80894485	-3.85272344
O	8.18773499	-13.87482626	-2.90329174
C	15.62297612	-14.91624692	-2.90889558
C	34.68131930	-13.11099123	-4.40119208

C	12.84881843	-17.53043109	-2.45168369
O	6.69896635	-12.27856349	-4.87699306
C	7.35567896	-10.64795516	-3.20685069
C	5.58696475	-11.47920676	-5.26649224
H	22.61093543	-8.06361849	-1.86640439
H	18.57767296	-5.91287756	-1.76498116
H	17.53035779	-8.33834468	-5.17860165
H	24.57084546	-8.65233303	-2.58355004
H	24.01303311	-10.35265745	-5.15730278
H	15.33528190	-7.80775303	-2.36814058
H	26.66606904	-10.57029700	-3.60050003
H	26.64282827	-7.71473712	-3.36299800
H	23.82882602	-12.47475776	-4.57907668
H	26.46088048	-8.95243378	-6.23214640
H	14.00048250	-9.72469970	-5.46622082
H	13.71942751	-6.87546415	-5.48538437
H	12.13702885	-8.40035599	-4.38355931
H	24.85489384	-14.50742193	-1.42589706
H	23.82526081	-15.08309771	-4.30498578
H	28.98797146	-11.00499793	-3.65986215
H	30.74032093	-14.21274120	-4.97738979
H	31.17840153	-11.15513318	-2.76868253
H	32.30486092	-12.25132012	-5.45515583
H	21.92095903	-10.74653840	-3.53867114
H	20.83608788	-7.33684080	-5.18544082
H	19.23498905	-7.19234245	0.34789043
H	20.77754318	-7.64272962	-0.38004972
H	22.65176581	-3.24252153	-3.85273119
H	20.33692120	-2.56828364	-4.60772351
H	20.28054991	-13.77576697	-6.08647689
H	20.91474613	-12.58012212	-7.27294454
H	22.04783393	-13.76065914	-6.49643884
H	16.14253751	-9.95181569	-4.51011729
H	15.50592279	-10.23917684	-2.90422573
H	27.12610149	-11.37903341	-5.79681819
H	26.33202955	-6.48024145	-6.20632331
H	26.11790313	-5.58147647	-4.71216136
H	27.71435839	-6.24308169	-5.10738927
H	25.91078386	-5.93561581	-1.12637143
H	24.68416929	-7.08979944	-0.59121242
H	24.48898482	-6.23661911	-2.14794322
H	26.03988989	-13.23452494	-3.92781750
H	25.88820665	-12.55752786	-2.29705658
H	28.71986706	-8.33382291	-5.41941307
H	14.13585127	-4.99963736	-3.82399200
H	12.47928632	-5.62442748	-3.63371950
H	13.73478395	-6.06978855	-2.47673582
H	21.99439786	-14.22399170	-1.95685793
H	21.78364838	-13.95868100	-3.72683334
H	12.79725752	-9.18508075	-2.20784588
H	23.57801521	-16.53096127	-1.78256334
H	27.67385676	-14.40072145	-2.60543164
H	27.08412371	-15.36218952	-1.19525762
H	27.74066146	-16.19590016	-2.64677394
H	28.58289586	-12.90863878	-5.31355903
H	29.86873202	-12.29645131	-6.38231083
H	14.30316652	-12.92010949	-3.64298737

```
  H    23.07452605    -17.05989454     -4.66235309
  H     9.87437937    -15.17165243     -3.19476454
  H     9.90098859    -11.66634112     -4.44861740
  H    25.41797988    -17.49726786     -3.76625756
  H    24.76022968    -18.60921216     -2.56446785
  H    24.62708374    -18.99411047     -4.28179937
  H    22.05533001    -19.24970500     -3.94559573
  H    22.19252336    -18.78899570     -2.24153104
  H    21.12235575    -17.88052129     -3.30672411
  H    30.47620159    -13.97169829     -2.68758901
  H    33.33658757    -10.05783864     -4.72963226
  H    32.49833662     -9.08010300     -3.53163946
  H    33.56233344    -10.38867761     -2.99236836
  H    15.87854723    -14.52575172     -3.89971056
  H    15.66728164    -14.12971726     -2.14788219
  H    16.29060497    -15.74547009     -2.64177621
  H    34.63425403    -13.54237750     -5.40641107
  H    34.92577195    -12.04655415     -4.48772047
  H    35.50207708    -13.60239181     -3.86097212
  H    13.48821404    -17.63961019     -3.33414848
  H    13.42077011    -17.17985529     -1.58611687
  H    12.33586421    -18.47499747     -2.22366441
  H     6.61378782     -9.89551932     -3.40578311
  H     8.02062014    -10.36012336     -2.40769759
  H     5.91943740    -10.51302344     -5.65767293
  H     5.13746082    -12.08844394     -6.06278376
  H     4.88595313    -11.35298922     -4.43588756
```

The last file will contain energies, orbitals, charges, etc. produced by GAUSSIAN (Espera.log) or ORCA (Espera.OUT)

5.5. FILE espera.log -------------------- follows with final coordinates and Gaussian archive

```
%nproc=24
%Mem=48GB
%chk=espr
# OPT FREQ ONIOM(wB97XD/6-31G**:PM7)

ESPERAMYCIN  -- ONIOM

0,1
      1          1           0        3.635420   -7.624491    4.362382
      2          8           0        3.014602   -7.805933    3.629431
      3          6           0        3.712085   -8.113620    2.440012
      4          1           0        2.891190   -8.386602    1.743789
      5          1           0        4.355697   -8.992821    2.613478
      6          6           0        4.511063   -6.929984    1.893798
```

7	1	0	5.304108	-7.262139	1.186417
8	8	0	3.619085	-6.153289	1.083991
9	6	0	3.577501	-4.826263	1.592065
10	1	0	4.308723	-4.221762	0.983054
11	7	0	2.241214	-4.313153	1.379834
12	6	0	1.016784	-4.908472	1.619205
13	1	0	0.935349	-5.942078	1.932534
14	7	0	0.024280	-4.082878	1.408370
15	6	0	0.622218	-2.897499	1.023827
16	6	0	0.056974	-1.619273	0.756212
17	8	0	-1.132007	-1.293959	0.792411
18	7	0	1.034445	-0.665543	0.469735
19	1	0	0.689629	0.289068	0.305092
20	6	0	2.383747	-0.895802	0.503472
21	7	0	3.214412	0.155006	0.283060
22	1	0	2.868885	1.116187	0.283528
23	1	0	4.109216	0.031004	0.742565
24	7	0	2.918440	-2.070704	0.738436
25	6	0	1.998333	-3.012202	1.001926
26	6	0	5.082588	-5.978140	2.998109
27	1	0	5.273109	-6.487533	3.960622
28	6	0	3.958982	-4.916707	3.076591
29	1	0	3.124646	-5.270140	3.702859
30	1	0	4.287265	-3.954849	3.499598
31	8	0	6.259527	-5.496761	2.392236
32	15	0	7.343201	-4.464498	3.073837
33	8	0	8.302554	-4.308193	1.961150
34	8	0	7.614231	-4.775250	4.486372
35	8	0	6.378089	-3.128302	3.212707
36	6	0	6.169158	-2.180845	2.188411
37	1	0	5.064807	-2.088529	2.091532
38	1	0	6.561131	-2.439924	1.185890
39	6	0	6.798955	-0.863865	2.690644
40	1	0	7.775114	-0.655630	2.200178
41	8	0	5.941505	0.206884	2.261505
42	6	0	5.466351	0.972078	3.346752
43	1	0	5.577975	2.040995	3.029442
44	7	0	4.052283	0.656174	3.502010
45	6	0	3.507800	-0.613071	3.670528
46	1	0	4.134027	-1.491459	3.802898
47	7	0	2.207514	-0.598309	3.633697
48	6	0	1.873789	0.726229	3.425719
49	6	0	0.594304	1.316382	3.231298
50	8	0	-0.502199	0.752692	3.207089
51	7	0	0.681377	2.693809	3.043629
52	1	0	-0.220001	3.168913	2.877873
53	6	0	1.849695	3.411807	3.039288
54	7	0	1.737178	4.739116	2.867072
55	1	0	0.839550	5.223163	2.936909
56	1	0	2.575456	5.281053	3.021220
57	7	0	3.048558	2.864206	3.160549
58	6	0	2.993053	1.533979	3.335241
59	6	0	6.934612	-0.758395	4.225658
60	1	0	6.502706	-1.618134	4.782604
61	6	0	6.269692	0.582551	4.591338
62	1	0	5.655522	0.510310	5.501084
63	1	0	7.051257	1.336013	4.817693

64	8	0	8.292515	-0.618242	4.580846
65	1	0	8.749434	-1.487797	4.518964
66	1	0	4.514611	4.554335	2.463982
67	8	0	4.592540	5.415119	2.945778
68	6	0	5.607487	6.220353	2.405840
69	1	0	6.432891	5.591012	2.022879
70	1	0	5.963005	6.807192	3.278952
71	6	0	5.067700	7.158314	1.322377
72	1	0	5.830258	7.928235	1.054259
73	8	0	4.893196	6.403840	0.120250
74	6	0	3.590271	6.543778	-0.407334
75	1	0	3.745593	6.781663	-1.490412
76	7	0	2.930948	5.246417	-0.295417
77	6	0	3.565708	4.036681	-0.064045
78	1	0	4.645627	3.961111	0.035850
79	7	0	2.715673	3.046091	-0.047161
80	6	0	1.481263	3.620164	-0.265437
81	6	0	0.185197	3.024991	-0.292216
82	8	0	-0.102116	1.841349	-0.080758
83	7	0	-0.810463	3.955361	-0.567689
84	1	0	-1.777591	3.573648	-0.647171
85	6	0	-0.602618	5.298000	-0.723441
86	7	0	-1.686312	6.064501	-0.933569
87	1	0	-2.575932	5.691976	-1.298982
88	1	0	-1.492354	7.038075	-1.094070
89	7	0	0.589362	5.873842	-0.648792
90	6	0	1.574916	4.990256	-0.422874
91	6	0	3.710184	7.817052	1.664310
92	1	0	3.239915	7.349871	2.559010
93	6	0	2.867540	7.638156	0.385854
94	1	0	1.816269	7.377399	0.616431
95	1	0	2.834027	8.573017	-0.205134
96	8	0	3.980903	9.219524	1.753508
97	15	0	3.533333	10.154155	2.967334
98	8	0	4.136402	11.446534	2.694047
99	8	0	3.131941	9.471216	4.185468
100	8	0	1.864871	10.571540	2.450484
101	6	0	0.998651	10.246478	1.642994
102	1	0	1.126656	9.518967	0.828250
103	6	0	-0.351759	10.911590	1.789794
104	1	0	-0.369786	11.886070	1.240760
105	8	0	-1.296231	10.093462	1.124214
106	6	0	-2.237350	9.536875	2.040218
107	1	0	-3.217741	9.603446	1.493096
108	7	0	-1.889164	8.156132	2.260318
109	6	0	-0.695555	7.661499	2.785783
110	1	0	0.100698	8.307845	3.122769
111	7	0	-0.689651	6.365232	2.842049
112	6	0	-1.913459	5.972073	2.345169
113	6	0	-2.449005	4.661378	2.166929
114	8	0	-1.890577	3.586762	2.409065
115	7	0	-3.745118	4.693588	1.662539
116	1	0	-4.214000	3.770423	1.587372
117	6	0	-4.420729	5.833371	1.306456
118	7	0	-5.649114	5.692493	0.814898
119	1	0	-6.164378	4.796650	0.769938
120	1	0	-6.112228	6.541255	0.538397

121	7	0	-3.911073	7.062084	1.438450
122	6	0	-2.686690	7.053928	1.960848
123	6	0	-0.808740	11.097647	3.260209
124	1	0	-0.070141	10.727400	4.002357
125	6	0	-2.159388	10.373405	3.329187
126	1	0	-2.268790	9.762581	4.241593
127	1	0	-2.985645	11.114130	3.370237
128	8	0	-1.069981	12.461363	3.490508
129	1	0	-0.262912	12.924837	3.799940
130	1	0	-10.158143	-1.086766	1.205905
131	8	0	-10.405603	-1.506526	0.342127
132	6	0	-10.477127	-0.512772	-0.661127
133	1	0	-10.842394	-1.047418	-1.557978
134	1	0	-9.454445	-0.125559	-0.827924
135	6	0	-11.445467	0.589106	-0.240052
136	1	0	-12.498507	0.249226	-0.216121
137	8	0	-11.129151	0.907671	1.131165
138	6	0	-10.784352	2.256875	1.264257
139	1	0	-11.145493	2.544274	2.278902
140	7	0	-9.312323	2.391284	1.232916
141	6	0	-8.646633	3.521602	0.786835
142	1	0	-9.165797	4.371545	0.366108
143	7	0	-7.356573	3.433629	0.932476
144	6	0	-7.153605	2.187896	1.482377
145	6	0	-5.938378	1.595540	1.908163
146	8	0	-4.813272	2.117665	1.876402
147	7	0	-6.118624	0.317419	2.408067
148	1	0	-5.260651	-0.145957	2.751258
149	6	0	-7.342835	-0.309485	2.506659
150	7	0	-7.344905	-1.558679	2.979815
151	1	0	-6.515542	-2.028182	3.367836
152	1	0	-8.248254	-1.994728	3.079740
153	7	0	-8.490147	0.262310	2.164443
154	6	0	-8.344692	1.497370	1.668552
155	6	0	-11.254739	1.903962	-1.052966
156	1	0	-10.217293	1.945577	-1.466492
157	6	0	-11.416308	2.980632	0.076033
158	1	0	-10.983685	3.942169	-0.208889
159	1	0	-12.491469	3.173372	0.245548
160	8	0	-12.306420	2.056115	-1.945090
161	15	0	-12.063894	2.452362	-3.537643
162	8	0	-13.001592	3.566882	-3.754635
163	8	0	-11.991119	1.192955	-4.308195
164	8	0	-10.479351	2.885154	-3.566223
165	6	0	-9.914192	4.074569	-3.032647
166	1	0	-9.120142	3.717101	-2.339153
167	1	0	-10.593364	4.749215	-2.485717
168	6	0	-9.281907	4.802719	-4.231371
169	1	0	-10.005672	5.462380	-4.753102
170	8	0	-8.284754	5.703933	-3.713237
171	6	0	-7.039465	5.509355	-4.332638
172	1	0	-6.756063	6.510603	-4.738650
173	7	0	-6.061477	5.143301	-3.303916
174	6	0	-4.908429	5.852538	-3.010914
175	1	0	-4.716679	6.830461	-3.425139
176	7	0	-4.115088	5.207634	-2.201457
177	6	0	-4.764494	4.016500	-1.944871

178	6	0	-4.317820	2.867222	-1.242709
179	8	0	-3.220131	2.720688	-0.676551
180	7	0	-5.246104	1.842954	-1.250990
181	1	0	-4.933903	0.944783	-0.850084
182	6	0	-6.476041	1.917306	-1.866086
183	7	0	-7.306785	0.879066	-1.749327
184	1	0	-7.043034	-0.013563	-1.313467
185	1	0	-8.020328	0.858883	-2.471745
186	7	0	-6.875449	2.967462	-2.564736
187	6	0	-5.985697	3.949198	-2.602488
188	6	0	-8.586999	3.840361	-5.214834
189	1	0	-8.582921	2.777266	-4.878341
190	6	0	-7.171737	4.412990	-5.401267
191	1	0	-6.413293	3.609745	-5.300643
192	1	0	-7.054133	4.815811	-6.421990
193	8	0	-9.216202	3.929798	-6.479092
194	1	0	-10.060536	3.426981	-6.459640
195	1	0	-11.291752	-3.446504	0.522482
196	8	0	-11.876691	-4.234160	0.621204
197	6	0	-11.402423	-5.309606	-0.154291
198	1	0	-11.265041	-4.980837	-1.200635
199	1	0	-12.235789	-6.035791	-0.100364
200	6	0	-10.116269	-5.913072	0.407111
201	1	0	-10.006447	-6.983602	0.118734
202	8	0	-9.018376	-5.246121	-0.237067
203	6	0	-8.073137	-4.889050	0.767455
204	1	0	-7.364352	-5.754481	0.895291
205	7	0	-7.348096	-3.733815	0.277503
206	6	0	-7.849949	-2.621825	-0.365199
207	1	0	-8.892139	-2.566256	-0.664359
208	7	0	-6.933832	-1.712415	-0.563725
209	6	0	-5.779403	-2.249769	-0.025041
210	6	0	-4.477075	-1.704949	0.088442
211	8	0	-4.095679	-0.573692	-0.255442
212	7	0	-3.581718	-2.601507	0.667674
213	1	0	-2.617789	-2.252514	0.767642
214	6	0	-3.921708	-3.845039	1.155443
215	7	0	-2.943878	-4.591560	1.696355
216	1	0	-1.975003	-4.292280	1.782112
217	1	0	-3.243235	-5.440648	2.155703
218	7	0	-5.139198	-4.337778	1.094954
219	6	0	-6.007298	-3.510294	0.499899
220	6	0	-9.941298	-5.735984	1.954857
221	1	0	-10.891146	-5.488980	2.469667
222	6	0	-8.900401	-4.593183	2.026983
223	1	0	-9.401168	-3.613804	1.980642
224	1	0	-8.293821	-4.611172	2.948283
225	8	0	-9.451501	-7.010403	2.312714
226	15	0	-8.920382	-7.464708	3.793350
227	8	0	-8.715230	-8.913847	3.591415
228	8	0	-9.634823	-6.798427	4.901247
229	8	0	-7.472313	-6.654578	3.857434
230	6	0	-6.305864	-7.031751	3.159292
231	1	0	-5.925229	-6.079529	2.720663
232	1	0	-6.451530	-7.743417	2.322952
233	6	0	-5.278675	-7.607215	4.161439
234	1	0	-5.012897	-8.658137	3.919440

235	8	0	-4.041462	-6.895836	3.956885
236	6	0	-3.651292	-6.188448	5.101740
237	1	0	-2.540172	-6.309083	5.166537
238	7	0	-3.963959	-4.769660	4.862855
239	6	0	-5.198714	-4.245824	4.545891
240	1	0	-6.110949	-4.838626	4.611381
241	7	0	-5.129084	-3.002717	4.160979
242	6	0	-3.780245	-2.693988	4.209767
243	6	0	-3.090612	-1.546481	3.746943
244	8	0	-3.569905	-0.522601	3.238849
245	7	0	-1.711162	-1.675159	3.891335
246	1	0	-1.155870	-0.859227	3.603537
247	6	0	-1.070430	-2.796003	4.359611
248	7	0	0.270776	-2.739004	4.452341
249	1	0	0.836457	-1.980294	4.071218
250	1	0	0.722268	-3.608150	4.681102
251	7	0	-1.700202	-3.892586	4.734741
252	6	0	-3.037909	-3.787172	4.625724
253	6	0	-5.640215	-7.466328	5.655342
254	1	0	-6.600641	-6.941655	5.854216
255	6	0	-4.438924	-6.740271	6.294019
256	1	0	-4.751852	-5.964879	7.007888
257	1	0	-3.832272	-7.462133	6.873907
258	8	0	-5.687768	-8.745997	6.254571
259	1	0	-6.530820	-9.192424	6.012789
260	19	0	-2.457873	0.994568	1.252193
261	6	0	5.178807	-3.548036	-3.257903
262	6	0	4.969279	-4.902331	-2.657282
263	6	0	4.076954	-2.785425	-3.427618
264	6	0	6.503599	-3.072942	-3.853084
265	6	0	3.771683	-5.656514	-3.312840
266	6	0	5.618493	-5.558377	-1.682682
267	6	0	2.765841	-3.304277	-2.950912
268	7	0	4.014941	-1.570778	-4.177252
269	8	0	7.564999	-3.239480	-2.878244
270	6	0	6.761083	-3.825042	-5.082504
271	6	0	2.488016	-4.726478	-3.394989
272	6	0	4.213191	-6.034365	-4.669304
273	8	0	3.439838	-6.835261	-2.586873
274	6	0	6.776212	-5.146672	-0.845578
275	8	0	1.947017	-2.598188	-2.354200
276	6	0	3.209704	-1.618808	-5.293523
277	6	0	8.457335	-2.127796	-2.647641
278	6	0	6.784018	-4.515368	-6.081065
279	8	0	1.439959	-5.332706	-2.643362
280	6	0	4.704079	-6.274353	-5.752611
281	16	0	7.874808	-6.591694	-0.316318
282	8	0	3.128756	-0.506668	-6.082229
283	8	0	2.571516	-2.625961	-5.619039
284	6	0	7.850029	-1.167512	-1.615519
285	8	0	9.689618	-2.661152	-2.220657
286	6	0	6.527682	-5.477032	-7.098996
287	6	0	0.173676	-5.573948	-3.369925
288	6	0	5.493285	-6.339842	-6.933024
289	16	0	8.311629	-7.634215	-2.215242
290	6	0	3.869695	0.701219	-5.794938
291	8	0	6.915185	-0.382743	-2.394118

292	6	0	8.966440	-0.296965	-1.032330
293	6	0	9.903761	-2.648885	-0.763093
294	6	0	-0.574461	-4.246477	-3.502256
295	8	0	-0.527044	-6.546912	-2.647259
296	16	0	10.266923	-6.868299	-2.915402
297	6	0	5.769765	0.252214	-1.783574
298	6	0	10.043424	-1.174011	-0.325876
299	8	0	9.569567	0.428660	-2.114581
300	6	0	11.080277	-3.555042	-0.476671
301	6	0	-0.903987	-3.799910	-2.087233
302	6	0	-1.042939	-6.136584	-1.328539
303	6	0	9.716848	-5.498849	-4.079710
304	6	0	6.101391	1.306297	-0.737519
305	8	0	5.097836	0.844769	-2.906202
306	7	0	11.418913	-0.728341	-0.580826
307	8	0	-1.320043	-2.387136	-2.045232
308	6	0	-1.823781	-4.817863	-1.419517
309	6	0	-1.891358	-7.286128	-0.831549
310	6	0	6.991658	2.439669	-1.258088
311	6	0	5.336922	2.273292	-3.125177
312	8	0	11.618530	0.688995	-0.224010
313	6	0	-2.434728	-1.852268	-2.577273
314	8	0	-3.034246	-5.078158	-2.132051
315	6	0	6.768414	2.674484	-2.773873
316	8	0	6.618453	3.603430	-0.480561
317	6	0	11.810155	0.870178	1.177413
318	6	0	-2.267571	-0.451965	-2.971318
319	8	0	-3.490590	-2.513410	-2.734077
320	7	0	7.770612	1.965214	-3.582028
321	6	0	7.545843	4.709550	-0.538062
322	6	0	12.723872	2.091384	1.351432
323	8	0	10.548249	1.094956	1.835867
324	6	0	-3.307725	0.287293	-3.587419
325	6	0	-0.994576	0.123670	-2.827629
326	6	0	8.677339	2.756163	-4.435150
327	6	0	12.058868	3.167262	2.216859
328	6	0	9.843369	2.289351	1.371483
329	6	0	-3.012151	1.565060	-4.085059
330	7	0	-4.581600	-0.283276	-3.699897
331	6	0	-0.714354	1.383790	-3.318897
332	6	0	9.678642	3.520610	-3.560094
333	6	0	9.398486	1.789389	-5.378926
334	6	0	10.721010	3.564625	1.600638
335	8	0	12.893171	4.352419	2.245762
336	6	0	8.506276	2.246669	2.082592
337	6	0	-1.733488	2.097056	-3.978193
338	6	0	-5.665805	0.199989	-4.389476
339	8	0	0.538990	1.972688	-3.244820
340	16	0	9.835272	4.896202	2.609215
341	8	0	-1.496492	3.279100	-4.649858
342	6	0	-6.843755	-0.732796	-4.410803
343	8	0	-5.702487	1.307356	-4.953480
344	6	0	1.637577	1.153816	-2.786633
345	6	0	10.633330	6.415808	1.871202
346	6	0	-0.962438	4.415780	-3.918264
347	8	0	-8.013520	-0.016379	-4.451294
348	6	0	-6.740649	-2.067717	-4.418721

349	6	0	-9.258287	-0.728320	-4.629352
350	1	0	6.407870	-2.008045	-4.079360
351	1	0	5.186829	-6.528503	-1.439003
352	1	0	4.397772	-0.698951	-3.812700
353	1	0	2.203230	-4.624060	-4.448285
354	1	0	2.529710	-6.712403	-2.228510
355	1	0	7.386287	-4.381008	-1.307331
356	1	0	6.419200	-4.790608	0.140625
357	1	0	8.631214	-1.591185	-3.580240
358	1	0	7.312627	-1.733387	-0.838628
359	1	0	7.140541	-5.509738	-7.989036
360	1	0	0.412765	-6.016874	-4.337856
361	1	0	5.267971	-7.077223	-7.692294
362	1	0	3.738430	1.326599	-6.673001
363	1	0	3.451369	1.198480	-4.917572
364	1	0	4.931665	0.486828	-5.638036
365	1	0	8.558623	0.396371	-0.288820
366	1	0	9.013222	-3.078373	-0.271061
367	1	0	0.048923	-3.508974	-4.012431
368	1	0	-1.487096	-4.415231	-4.078745
369	1	0	5.094943	-0.513325	-1.384964
370	1	0	9.880287	-1.126765	0.756626
371	1	0	8.886997	0.971222	-2.607242
372	1	0	11.257091	-3.582965	0.604580
373	1	0	11.985153	-3.209577	-0.970246
374	1	0	10.838765	-4.567792	-0.810970
375	1	0	0.023244	-3.740651	-1.517497
376	1	0	-0.177071	-5.974067	-0.675392
377	1	0	9.146479	-4.756152	-3.523682
378	1	0	9.125769	-5.924022	-4.887647
379	1	0	10.635233	-5.052446	-4.461117
380	1	0	5.147914	1.745671	-0.428010
381	1	0	6.531327	0.842615	0.160159
382	1	0	11.578415	-0.684866	-1.586303
383	1	0	-2.020959	-4.497870	-0.394126
384	1	0	-1.304763	-8.206480	-0.806749
385	1	0	-2.754506	-7.412028	-1.484996
386	1	0	-2.252585	-7.061726	0.180292
387	1	0	8.055901	2.240207	-1.102505
388	1	0	4.629498	2.845967	-2.514604
389	1	0	5.125464	2.446573	-4.178946
390	1	0	-3.482442	-4.228328	-2.334790
391	1	0	6.854018	3.749067	-2.973319
392	1	0	12.196652	-0.049231	1.617822
393	1	0	7.382844	1.147860	-4.041834
394	1	0	7.205516	5.428545	0.205928
395	1	0	7.544898	5.189286	-1.522782
396	1	0	8.560366	4.381643	-0.289006
397	1	0	12.930569	2.520739	0.367499
398	1	0	13.679229	1.790484	1.789126
399	1	0	-0.228224	-0.475314	-2.356025
400	1	0	8.101770	3.482005	-5.036418
401	1	0	11.889137	2.798080	3.235550
402	1	0	9.704758	2.178489	0.288951
403	1	0	-3.769853	2.136971	-4.597095
404	1	0	-4.671933	-1.184727	-3.237665
405	1	0	10.366045	4.100598	-4.180576

406	1	0	10.253946	2.813078	-2.952378
407	1	0	9.169666	4.218130	-2.885006
408	1	0	8.686809	1.260655	-6.023667
409	1	0	10.100722	2.327020	-6.018864
410	1	0	9.956389	1.049416	-4.793542
411	1	0	10.936276	4.031645	0.633123
412	1	0	13.641341	4.232844	2.849226
413	1	0	7.810922	2.992976	1.698608
414	1	0	8.627477	2.378473	3.160994
415	1	0	8.070077	1.248656	1.924184
416	1	0	2.513366	1.796656	-2.816698
417	1	0	1.481121	0.819513	-1.760135
418	1	0	1.773987	0.278835	-3.430549
419	1	0	11.711946	6.310367	1.948343
420	1	0	10.282612	7.269247	2.450518
421	1	0	10.331137	6.528912	0.827470
422	1	0	-0.730128	5.159528	-4.677363
423	1	0	-1.730741	4.803467	-3.244867
424	1	0	-0.061236	4.139427	-3.368889
425	1	0	-5.776825	-2.555679	-4.406767
426	1	0	-7.607594	-2.716863	-4.441447
427	1	0	-9.461274	-1.383391	-3.773706
428	1	0	-9.245978	-1.312470	-5.551012
429	1	0	-10.022771	0.052838	-4.688883

Rotational constants (GHZ): 0.0042784 0.0026709 0.0020978

.5. Test job not archived.

1\1\GINC-SL054\FOpt\ONIOM(wB97XD/6-31G(d,p):wB97XD/6-31G:PM7)\Mixed\C1
39H175K1N44O62P4S4(2-)\ROOT\17-Oct-2025\0\\# opt=loose oniom(wb97xd/6-
31g(d,p):wb97xd/6-31g:pm7) iop(1/8=1,5/7=6144)\\ONIOM-separation of th
e antiparallel stem-Q2: coordinates from Sponer, JACS 2013, 135, 9785-
9796 + esperamycin\\-2,1\H,1.5305624852,-8.9455178525,2.4761479613\O,0
.9200467314,-8.8173707051,1.7235505503\C,1.5838837654,-9.0208638177,0.
4931926419\H,0.7561313028,-8.9405449889,-0.2426671677\H,1.999953907,-1
0.0424563272,0.4696108652\C,2.6577426506,-7.9690124001,0.2136494805\H,
3.3860963536,-8.3200734701,-0.5517865756\O,2.0067923469,-6.8519445906,
-0.4052939657\C,2.2464435589,-5.6904432206,0.3785082454\H,3.1246820877
,-5.1590197852,-0.0876323946\N,1.07486711,-4.8459943297,0.2874022141\C
,-0.263311569,-5.1751053757,0.3964749779\H,-0.5941564005,-6.203184292,
0.4785907625\N,-1.0291065433,-4.114850766,0.373222174\C,-0.1579492682,
-3.0485185503,0.2526120501\C,-0.4019360764,-1.646687118,0.2704962256\O
,-1.4850752802,-1.0674735987,0.380668271\N,0.7804977051,-0.9097192993,
0.1940278303\H,0.6717926762,0.1116613212,0.2414849679\C,2.0379833232,-
1.4510360111,0.1721522116\N,3.0968104633,-0.6015790336,0.181753458\H,2
.9811149292,0.3912672486,0.391821503\H,3.9166376723,-1.0258189638,0.60
02017289\N,2.2774886387,-2.7407626332,0.1449016921\C,1.1547161721,-3.4
746795527,0.201373836\C,3.3793236172,-7.4331325822,1.495953987\H,3.402
3251908,-8.1647456806,2.3244602486\C,2.526433871,-6.1796233365,1.80673
5523\H,1.6046623819,-6.4523243845,2.3445211055\H,3.0460253426,-5.43317
88536,2.4269940105\O,4.6625436405,-7.1234857989,1.0045692832\P,5.92046
67339,-6.5416733734,1.8897922007\O,6.9416056439,-6.3823647277,0.833970
6301\O,6.0459900802,-7.198841722,3.2003847161\O,5.2817814897,-5.077287
417,2.3185105665\C,5.3441870558,-3.9125956895,1.5247174302\H,4.2962269
187,-3.5461264711,1.4541622857\H,5.7132995677,-4.0380319105,0.48845769
86\C,6.2345568381,-2.9164634849,2.2982040991\H,7.2545219323,-2.8434617

265,1.8610001889\O,5.6665660022,-1.6089770521,2.114491267\C,5.32865154
33,-1.0013090623,3.3415237482\H,5.6971276146,0.0540574737,3.2630663827
\N,3.8741053236,-1.0031073624,3.4297410633\C,3.0459132057,-2.115822446
4,3.3210906992\H,3.4472094473,-3.1238804264,3.2576292651\N,1.786846266
6,-1.7898129199,3.2930540997\C,1.7757453695,-0.4108179137,3.3783563597
\C,0.6762062772,0.4896834855,3.3211318165\O,-0.5178875505,0.2164273778
,3.1792853565\N,1.0853826016,1.8161226754,3.4361974734\H,0.3257357836,
2.5129238222,3.3806437423\C,2.3860088882,2.2250480262,3.5833619703\N,2
.588919671,3.5473540505,3.7033734898\H,1.8238319012,4.2019155734,3.879
7346395\H,3.5208303168,3.8328878461,3.9682893346\N,3.4203735528,1.3992
685105,3.5786830257\C,3.0533186134,0.1129798717,3.4610660784\C,6.31805
61162,-3.1729036148,3.8190785265\H,5.6747959289,-4.0055445027,4.177978
0393\C,5.961596483,-1.8222317517,4.4691538102\H,5.3049159117,-1.939585
3264,5.3438307736\H,6.8834273705,-1.3379815741,4.85061784\O,7.65359199
06,-3.4327468325,4.191272518\H,7.9014863181,-4.3517116918,3.9407087929
\H,5.2659641699,2.8075010004,3.2597362304\O,5.5162098643,3.5041652483,
3.9163838348\C,6.7130328553,4.1450795458,3.5601565858\H,7.3894084276,3
.4357913324,3.0468849796\H,7.1518872956,4.4340439959,4.5384225186\C,6.
4543871746,5.3906837141,2.7077455753\H,7.3848850201,5.9996228455,2.610
1349856\O,6.1686592029,4.9699089334,1.3712953921\C,4.9589100586,5.5191
580814,0.8913218959\H,5.2156323056,5.937722774,-0.1149926214\N,4.01529
83726,4.4186047225,0.7217387695\C,4.3442796988,3.0731717724,0.68306748
62\H,5.3721814398,2.727671184,0.7602855265\N,3.2900416414,2.3284533385
,0.4879029371\C,2.2317693775,3.2081976739,0.4037406994\C,0.8365585215,
2.9522984991,0.2532856487\O,0.2759835878,1.8515634384,0.2041763227\N,0
.0947603633,4.1263936487,0.1896551409\H,-0.9292354935,4.0072509379,0.0
328290109\C,0.6118751525,5.3848126558,0.3278995211\N,-0.2561531614,6.4
101754715,0.2928582937\H,-1.1891715014,6.3421173829,-0.1413876637\H,0.
1631104699,7.3226262501,0.3465140158\N,1.8991973372,5.6365914726,0.521
2746784\C,2.6442320382,4.5197406754,0.5467056217\C,5.2693471955,6.2610
394,3.1892676954\H,4.6627806489,5.7384257755,3.9631234915\C,4.46953292
,6.5589211329,1.9054977738\H,3.3767897264,6.5086398328,2.0778807044\H,
4.6791173871,7.578937518,1.5313395576\O,5.8497220366,7.5096992118,3.57
93784386\P,5.5713757129,8.2444947039,4.9685603558\O,6.4667816443,9.387
6241807,4.9797357717\O,4.9670422011,7.4325904012,6.0111359921\O,4.0693
083101,9.1402724766,4.5605358281\C,3.1908147934,9.2053173399,3.7049305
793\H,3.1870649217,8.6573900109,2.7513963462\C,2.0233673494,10.1213461
435,3.9973166571\H,2.2551040073,11.1664592244,3.6726472222\O,0.9490951
834,9.706778572,3.1736476391\C,-0.1367459022,9.2049738214,3.9506286039
\H,-1.0488034562,9.6132763664,3.4345072861\N,-0.1250381314,7.766694720
1,3.864923294\C,0.8972484883,6.9068832716,4.2663366216\H,1.8035838024,
7.2626413797,4.7325268061\N,0.6032789495,5.6635107543,4.0402484982\C,-
0.6531643456,5.6818737282,3.474401545\C,-1.4657746586,4.6011031389,3.0
182225729\O,-1.1805516537,3.3994714171,3.0196271866\N,-2.6946852536,5.
0416216952,2.5375152928\H,-3.3585481692,4.2911905839,2.2657212118\C,-3
.0735183413,6.3566183538,2.4394265237\N,-4.2768508123,6.6143260776,1.9
334790489\H,-4.9809764468,5.8942820532,1.6972645854\H,-4.5195377405,7.
5866060463,1.8492226232\N,-2.3026804423,7.3754344585,2.8327815616\C,-1
.1388480276,6.9707125894,3.3365438014\C,1.5522001238,10.0936966888,5.4
747495165\H,2.1503681875,9.4125263365,6.1163057523\C,0.0698184877,9.70
78268706,5.3899931465\H,-0.2196520851,8.9607312138,6.1486855166\H,-0.5
656994407,10.5953553707,5.59363359\O,1.5998305622,11.4001789976,5.9961
337504\H,2.4760780797,11.5857007409,6.3957457695\H,-10.2327179878,1.15
33594565,0.861996609\O,-10.5286686462,0.9956883557,-0.0713302353\C,-10
.3230555062,2.1677138336,-0.8350452156\H,-10.758305997,1.9355813399,-1
.8251356751\H,-9.2321444831,2.3311879078,-0.9176496256\C,-11.031915520
3,3.3507894587,-0.1816148143\H,-12.1347409785,3.2695627521,-0.22810883

88\O,-10.7161977941,3.2889562072,1.2248959678\C,-10.07807991,4.4604488
98,1.6460403529\H,-10.4112992094,4.6029804231,2.7001633206\N,-8.614631
2616,4.2502381302,1.6392394238\C,-7.6873113555,5.2614917184,1.44637324
88\H,-7.9774046736,6.2785210053,1.2217808451\N,-6.4601070915,4.8453783
278,1.564799027\C,-6.5742375335,3.4994094675,1.8308437438\C,-5.5486150
446,2.5628980095,2.1136869551\O,-4.3335912621,2.8018625116,2.191780306
7\N,-6.0403803856,1.2864283141,2.325294444\H,-5.3286332562,0.573368628
2,2.5567621901\C,-7.378928591,0.957080134,2.2900891739\N,-7.6895714251
,-0.3279652352,2.4812083533\H,-7.009163681,-1.0496312413,2.7552160459\
H,-8.672491197,-0.551551197,2.4875126547\N,-8.3471838888,1.8405095792,
2.0841256602\C,-7.8993015516,3.0834069112,1.8672274278\C,-10.506744466
8,4.725894911,-0.6908627436\H,-9.4690132499,4.6101660395,-1.0892972659
\C,-10.4704699544,5.5465145517,0.6452614846\H,-9.8160434094,6.41803200
21,0.5739805138\H,-11.4796303272,5.9450860808,0.8564274001\O,-11.45219
27262,5.3050869582,-1.5249691951\P,-11.0503145018,5.9613247953,-2.9946
296954\O,-11.6963240573,7.2842345697,-2.9614607423\O,-11.2317375428,4.
9122292882,-4.0201218032\O,-9.4092280291,6.0073565033,-2.9344629815\C,
-8.6124165713,6.8909273921,-2.1577966765\H,-7.9551073875,6.2192045024,
-1.5611674942\H,-9.1439883575,7.5743016581,-1.4751660003\C,-7.77415614
57,7.6876086594,-3.1724616499\H,-8.3020012908,8.593309474,-3.536161660
7\O,-6.6226560575,8.1998926744,-2.4749125205\C,-5.4272445418,7.8549637
072,-3.126279012\H,-4.9030731466,8.824696469,-3.3066206675\N,-4.608918
5556,7.0611303897,-2.2049239244\C,-3.3392012284,7.4024009344,-1.769332
6055\H,-2.9090935685,8.373179625,-1.9624157372\N,-2.7539313907,6.43354
70356,-1.1218045997\C,-3.6703975148,5.4008682402,-1.1271689011\C,-3.53
26815374,4.0571974853,-0.6924590882\O,-2.5258057216,3.5415438221,-0.17
5514914\N,-4.6695247081,3.3041520881,-0.9192072386\H,-4.5907643896,2.2
939696636,-0.7236675024\C,-5.8192049911,3.7926029452,-1.4990923202\N,-
6.8703286659,2.9770228165,-1.6071574311\H,-6.8390557267,1.9760396555,-
1.3761171911\H,-7.5345397967,3.2776603917,-2.3141789513\N,-5.933847593
2,5.0304113477,-1.9520321475\C,-4.8420372669,5.7618507461,-1.779301827
5\C,-7.2725819211,6.8198935874,-4.3436679874\H,-7.5281397674,5.7389242
547,-4.2456417847\C,-5.7565702306,7.0716265966,-4.4066721801\H,-5.2080
072401,6.1106584362,-4.4853149446\H,-5.5016706688,7.6423376404,-5.3162
247455\O,-7.804060496,7.3194691617,-5.5561851338\H,-8.7411002653,7.035
74368,-5.6431285315\H,-11.8433056516,-0.6760858837,-0.312581871\O,-12.
5971720881,-1.3075669926,-0.3848268773\C,-12.345858386,-2.2749792159,-
1.376724995\H,-12.0874874119,-1.7737451521,-2.3274128503\H,-13.3250505
763,-2.7805298985,-1.4784729032\C,-11.2602337505,-3.2673220034,-0.9641
572964\H,-11.3851488998,-4.247873606,-1.4781350085\O,-10.0095291901,-2
.7549810306,-1.4524219514\C,-9.0561891158,-2.8498256853,-0.3978195597\
H,-8.5714430472,-3.8639448246,-0.4631968982\N,-8.0633820165,-1.8195320
306,-0.6283265724\C,-8.2661020437,-0.5109165879,-1.0128731694\H,-9.252
4853651,-0.1510607722,-1.2890902024\N,-7.1576321549,0.1797388018,-1.01
28679577\C,-6.1838522932,-0.7142428559,-0.6076686967\C,-4.7981903021,-
0.5259354683,-0.3835090115\O,-4.1518897512,0.5310965684,-0.4753735273\
N,-4.1605227441,-1.7087502407,-0.0156517528\H,-3.1481020673,-1.6242425
074,0.1540930305\C,-4.7990974611,-2.9124568408,0.1921876132\N,-4.04505
24945,-3.9640809196,0.5548602345\H,-3.0385435822,-3.9248988821,0.69993
76501\H,-4.5523882454,-4.7969746007,0.8203112074\N,-6.0928464052,-3.08
2401943,0.0307428689\C,-6.7190954577,-1.9681640252,-0.367633594З\C,-11
.1227028967,-3.4676920058,0.5845290942\H,-12.0139163003,-3.1200037089,
1.1440849241\C,-9.8522273324,-2.6420211397,0.8988895612\H,-10.11247028
21,-1.5856818601,1.06776953\H,-9.310159072,-2.9959377981,1.7921754963\
O,-10.9554617943,-4.8673539787,0.6558391762\P,-10.6131854949,-5.736003
2177,2.0008505801\O,-10.7362646322,-7.1164262325,1.4888417626\O,-11.20
74814505,-5.1710085643,3.2294206204\O,-9.0226952201,-5.3195766933,2.23

37694129\C,-7.9420558001,-5.8026722701,1.4662383871\H,-7.3330434171,-4
.8953095234,1.243075648\H,-8.2072282122,-6.2669671843,0.496049391\C,-7
.1226264091,-6.8011123064,2.3160776409\H,-7.0936138017,-7.8093556107,1
.8510594468\O,-5.7470690403,-6.3722239052,2.2661063994\C,-5.2597452937
,-6.0344690565,3.5356574748\H,-4.2101764622,-6.4226065359,3.5687304945
\N,-5.2273134229,-4.5644229424,3.6111419192\C,-6.2927682025,-3.7113895
122,3.419750851\H,-7.3186216225,-4.0743606563,3.3584905511\N,-5.922008
101,-2.4665976581,3.3132114488\C,-4.5421701741,-2.4995206187,3.4228682
942\C,-3.5868414366,-1.4740152754,3.2172962886\O,-3.7941815269,-0.2827
717838,2.944975095\N,-2.2819977919,-1.9494042985,3.3253539457\H,-1.541
5384797,-1.2441486849,3.2192577742\C,-1.9380385584,-3.2620063843,3.537
2002499\N,-0.6253849966,-3.5413191903,3.6352136339\H,0.1164738439,-2.8
730485254,3.4255686622\H,-0.3964122394,-4.5201224848,3.6684887097\N,-2
.8193521784,-4.2346371427,3.667985189\C,-4.0906154289,-3.798575875,3.5
89266121\C,-7.5124625311,-6.8989234135,3.8063016409\H,-8.3353959737,-6
.2184196717,4.1176647265\C,-6.2083280755,-6.6261179584,4.5828173883\H,
-6.3686270228,-5.9681478875,5.4489263453\H,-5.8113491368,-7.5757507416
,4.9902314369\O,-7.8802683041,-8.2289218285,4.1140548644\H,-8.79079096
4,-8.4041625347,3.7843103699\K,-2.2714357846,1.3171225239,1.330414314\
C,4.3254775719,-3.825923676,-4.084833761\C,3.7830262353,-5.189138109,-
3.7913102689\C,3.4369927944,-2.8085550316,-4.0812003419\C,5.7505016147
,-3.5590953324,-4.5676787135\C,2.4768369984,-5.4859260048,-4.590416509
5\C,4.2178099591,-6.1697881231,-2.9844593236\C,2.0208418905,-3.0950398
687,-3.7235472701\N,3.6904838811,-1.4828008985,-4.5494784764\O,6.69819
21488,-4.1717127196,-3.655977399\C,5.8866038441,-4.0729074167,-5.93182
66946\C,1.4458118222,-4.2856710962,-4.4643467641\C,2.8836227791,-5.660
7700551,-5.998126693\O,1.8497673068,-6.6804591051,-4.1360306458\C,5.39
8166257,-6.2270753786,-2.0822201478\O,1.3583467774,-2.3596881734,-2.98
49956972\C,2.9493598665,-1.1038021427,-5.6466889557\C,7.8096084834,-3.
3743439579,-3.1931155697\C,5.7979118632,-4.5220735311,-7.0563637301\O,
0.2523966703,-4.7747839211,-3.8582066706\C,3.3570980559,-5.7741379704,
-7.1094725401\S,6.1101088407,-7.9673277662,-1.8827756575\O,3.162797969
6,0.1373744624,-6.1752615612\O,2.1134784393,-1.8413535713,-6.180469684
9\C,7.3904282622,-2.5393876602,-1.9751761584\O,8.8652768318,-4.2591113
895,-2.8963758285\C,5.3764369911,-5.1592882614,-8.2576151059\C,-0.9996
726805,-4.5537262839,-4.6151886487\C,4.165188135,-5.7711631995,-8.2788
694838\S,6.3856662103,-8.6570340212,-3.9641089467\C,4.1463860276,1.049
4598675,-5.635656601\O,6.6981758816,-1.4112337012,-2.5617477089\C,8.64
77731268,-2.0978712248,-1.2211995717\C,9.0073863692,-4.6059713559,-1.4
715971185\C,-1.4165864352,-3.0909994223,-4.4538981047\O,-1.9380652785,
-5.4659840051,-4.1180788007\S,8.4952962258,-8.2395280357,-4.488682584\
C,5.7011960782,-0.6698997619,-1.8239276842\C,9.4604833347,-3.331580258
9,-0.7255749441\O,9.4515995341,-1.3214084979,-2.1226029334\C,9.9300500
959,-5.8017189957,-1.3926868086\C,-1.7015202134,-2.8895370465,-2.97453
17983\C,-2.4079466687,-5.2349325187,-2.7399045693\C,8.3293493276,-6.56
49505521,-5.3264439901\C,6.2157200848,0.0314499657,-0.5754604397\O,5.2
367767235,0.2871346526,-2.7889671625\N,10.9119711067,-3.1765222547,-0.
882844321\O,-1.7842630688,-1.4604401375,-2.6257706964\C,-2.860616553,
-3.7816071529,-2.5400233727\C,-3.5197348498,-6.2324509891,-2.500844929
9\C,7.3656037499,1.0088211244,-0.8411957027\C,5.8069345797,1.633121336
8,-2.6939377695\O,11.4139730749,-1.9537478771,-0.2280007355\C,-2.72028
6016,-0.5795221542,-3.0251682681\O,-4.0634469572,-3.5946563109,-3.2876
419543\C,7.2740189704,1.6045955549,-2.269217177\O,7.2326892655,2.03596
51195,0.1714626547\C,11.5755522261,-2.1231683329,1.1786697852\C,-2.218
2506852,0.7935557126,-3.1069013506\O,-3.8909318492,-0.9267074785,-3.31
77272581\N,8.1240641317,0.8680624177,-3.2155380178\C,8.3905296954,2.88
08118118,0.3517396604\C,12.7355281884,-1.2148989659,1.6099572079\O,10.

3690596249,-1.7539565561,1.8747684917\C,-3.0310007513,1.8687783966,-3.
5443231422\C,-0.8554744586,1.0115756036,-2.8464681395\C,9.2272014512,1
.5870049524,-3.8802037835\C,12.2946209093,-0.2214814242,2.6904347462\C
,9.9794034078,-0.3573744203,1.6828989509\C,-2.4272968418,3.1174514598,
-3.7542158663\N,-4.3949470496,1.6491673471,-3.7731868842\C,-0.27104205
42,2.2457466985,-3.0539091884\C,10.3345230415,1.8930146576,-2.86388373
96\C,9.7513993242,0.7005636716,-5.0137187271\C,11.1140894112,0.5989763
994,2.1798618467\O,13.3759856076,0.7018623268,2.972530261\C,8.63603100
07,-0.2355186289,2.3726947979\C,-1.0672688202,3.3005253324,-3.53921053
91\C,-5.305714446,2.507320033,-4.3376715368\O,1.0789595596,2.495678353
8,-2.8584859206\S,10.5104037268,1.8563915876,3.4563128596\O,-0.5342264
033,4.5088887381,-3.9395264717\C,-6.6637125363,1.9022578454,-4.5564177
097\O,-5.0609548463,3.6860672128,-4.6480817014\C,1.9377398608,1.364648
4592,-2.5926922049\C,11.6694246885,3.2678575976,3.0623651352\C,0.21089
89417,5.3077958449,-2.9808710345\O,-7.6349187097,2.864337579,-4.436401
3308\C,-6.8690020153,0.613044196,-4.8538949682\C,-8.9998616207,2.51763
80905,-4.7600367336\H,5.9121554303,-2.4782932981,-4.5573863549\H,3.564
2973601,-7.0409528945,-2.9553050505\H,4.2452408715,-0.8221846993,-4.00
59990526\H,1.2421921991,-3.8989925038,-5.4693565471\H,0.9761171978,-6.
4267699357,-3.7562561711\H,6.1885920894,-5.5455320914,-2.3692309441\H,
5.0860410675,-6.0143331858,-1.0409939698\H,8.1457140054,-2.7084844235,
-3.9878425887\H,6.7015447329,-3.1150236994,-1.3374782922\H,6.006861264
8,-5.1453863001,-9.1358003063\H,-0.8229661972,-4.8251735632,-5.6569792
704\H,3.8130432424,-6.2575323005,-9.1791067792\H,4.2035829016,1.859407
0817,-6.3567944335\H,3.8121491691,1.4335640103,-4.6698479022\H,5.12232
958,0.5643639369,-5.5328147774\H,8.3750135423,-1.5018390056,-0.3435978
109\H,8.0198883754,-4.9092929367,-1.0811372206\H,-0.6173855706,-2.4290
474984,-4.7943312131\H,-2.3153074236,-2.9156455188,-5.0499451067\H,4.8
508346193,-1.3228168195,-1.5982973663\H,9.2615312423,-3.477622338,0.34
2014345\H,8.9355988499,-0.5426161079,-2.4834570632\H,10.0444445791,-6.
0983602124,-0.3438468851\H,10.9123054177,-5.5811577294,-1.8029192923\H
,9.4789654065,-6.6355269584,-1.9376933743\H,-0.8133802849,-3.170781335
,-2.4088659961\H,-1.5597478156,-5.4214832714,-2.0702050481\H,7.9187241
619,-5.8443797277,-4.62057533\H,7.6954389878,-6.6591828321,-6.20518488
79\H,9.3425666953,-6.2754991012,-5.6053443614\H,5.3747663033,0.6059746
894,-0.1746096468\H,6.4850305569,-0.6990793813,0.1987927227\H,11.12453
80618,-2.9598385261,-1.8555610251\H,-3.0274637975,-3.6487795632,-1.468
9398339\H,-3.1615013184,-7.2482982845,-2.6782695138\H,-4.3568845454,-6
.0117248379,-3.162908888\H,-3.8673412031,-6.1490859323,-1.4631150406\H
,8.3472405354,0.5376391167,-0.736413618\H,5.2215185817,2.2128753252,-1
.971179968\H,5.6908675277,2.0699900079,-3.6843137248\H,-4.2948721265,-
2.6404608883,-3.2997128097\H,7.6129240263,2.6464881562,-2.2312912323\H
,11.7198111883,-3.1792440836,1.4078996634\H,7.5814926184,0.2804470274,
-3.8401795971\H,8.1892447706,3.4853014853,1.2351273915\H,8.5460954695,
3.5446283624,-0.5055620676\H,9.2899744586,2.2795964921,0.5201095558\H,
13.0813886139,-0.6476364996,0.7417491328\H,13.5747213344,-1.8165572682
,1.9685481065\H,-0.2699512273,0.164096828,-2.5187046351\H,8.8623651577
,2.5376662487,-4.3077394859\H,11.9968451993,-0.7476459124,3.6054842061
\H,9.8704075337,-0.2010992134,0.6025848523\H,-3.0087032199,3.945771315
5,-4.1273495519\H,-4.7111978678,0.7170725322,-3.517029229\H,11.1650841
359,2.4138552139,-3.3463773594\H,10.7029933444,0.9584261936,-2.4260826
771\H,9.9676065904,2.5311601013,-2.0518024697\H,8.9687916242,0.5017427
214,-5.7551566012\H,10.5875950672,1.1818567129,-5.5244413262\H,10.0965
72834,-0.2560127817,-4.6046878427\H,11.4761363885,1.1965024586,1.33581
39175\H,14.0474591401,0.2856703208,3.5330057845\H,8.1491538078,0.71661
27977,2.162145451\H,8.7330946412,-0.3669748162,3.4535789647\H,7.990726
1061,-1.0470105869,2.0032961518\H,2.9379511562,1.7761349162,-2.4858708

678\H,1.6604948408,0.866824262,-1.6625086463\H,1.9002931865,0.63866991
94,-3.41145922\H,12.6902898571,2.8991300549,3.1109006899\H,11.49662997
01,4.0372007388,3.8141769176\H,11.4508772433,3.6667255141,2.0690722465
\H,0.6430792728,6.1198367647,-3.5615345027\H,-0.4790669539,5.712956494
4,-2.2366622353\H,0.9977858975,4.7184713915,-2.5077384454\H,-6.0443183
135,-0.0780258216,-4.951499666\H,-7.8595642236,0.2046749318,-5.0136507
174\H,-9.3877760939,1.7624583196,-4.0660053599\H,-9.078189523,2.155442
7041,-5.7864466808\H,-9.5613016335,3.4503649513,-4.6460442629\\Version
=ES64L-G16RevC.01\HF=0.3999209\RMSD=4.705e-09\RMSF=2.698e-05\Dipole=21
.9437475,12.6862198,2.933676\PG=C01 [X(C139H175K1N44O62P4S4)]\\@

LAWS OF PROGRAMMING DEFINITION: A WORKING PROGRAM IS ONE THAT HAS ONLY UNOBSERVED BUGS.
